# Methods used to evaluate usability of mobile clinical decision support systems for healthcare emergencies: a systematic review and qualitative synthesis

**DOI:** 10.1093/jamiaopen/ooad051

**Published:** 2023-07-12

**Authors:** Jared M Wohlgemut, Erhan Pisirir, Evangelia Kyrimi, Rebecca S Stoner, William Marsh, Zane B Perkins, Nigel R M Tai

**Affiliations:** Centre for Trauma Sciences, Blizard Institute, Queen Mary University of London, London, UK; Trauma Service, Royal London Hospital, Barts NHS Health Trust, London, UK; Department of Electrical Engineering and Computer Science, Queen Mary University of London, London, UK; Department of Electrical Engineering and Computer Science, Queen Mary University of London, London, UK; Centre for Trauma Sciences, Blizard Institute, Queen Mary University of London, London, UK; Trauma Service, Royal London Hospital, Barts NHS Health Trust, London, UK; Department of Electrical Engineering and Computer Science, Queen Mary University of London, London, UK; Centre for Trauma Sciences, Blizard Institute, Queen Mary University of London, London, UK; Trauma Service, Royal London Hospital, Barts NHS Health Trust, London, UK; Centre for Trauma Sciences, Blizard Institute, Queen Mary University of London, London, UK; Trauma Service, Royal London Hospital, Barts NHS Health Trust, London, UK; Academic Department of Military Surgery and Trauma, Royal Centre of Defence Medicine, Birmingham, UK

**Keywords:** usability, mobile health, clinical decision support systems, healthcare emergencies, systematic review

## Abstract

**Objective:**

The aim of this study was to determine the methods and metrics used to evaluate the usability of mobile application Clinical Decision Support Systems (CDSSs) used in healthcare emergencies. Secondary aims were to describe the characteristics and usability of evaluated CDSSs.

**Materials and Methods:**

A systematic literature review was conducted using Pubmed/Medline, Embase, Scopus, and IEEE Xplore databases. Quantitative data were descriptively analyzed, and qualitative data were described and synthesized using inductive thematic analysis.

**Results:**

Twenty-three studies were included in the analysis. The usability metrics most frequently evaluated were efficiency and usefulness, followed by user errors, satisfaction, learnability, effectiveness, and memorability. Methods used to assess usability included questionnaires in 20 (87%) studies, user trials in 17 (74%), interviews in 6 (26%), and heuristic evaluations in 3 (13%). Most CDSS inputs consisted of manual input (18, 78%) rather than automatic input (2, 9%). Most CDSS outputs comprised a recommendation (18, 78%), with a minority advising a specific treatment (6, 26%), or a score, risk level or likelihood of diagnosis (6, 26%). Interviews and heuristic evaluations identified more usability-related barriers and facilitators to adoption than did questionnaires and user testing studies.

**Discussion:**

A wide range of metrics and methods are used to evaluate the usability of mobile CDSS in medical emergencies. Input of information into CDSS was predominantly manual, impeding usability. Studies employing both qualitative and quantitative methods to evaluate usability yielded more thorough results.

**Conclusion:**

When planning CDSS projects, developers should consider multiple methods to comprehensively evaluate usability.

## BACKGROUND

### Introduction

Clinical decision support systems (CDSSs) have been developed as potentially powerful diagnostic adjuncts in many clinical situations.^1^ A CDSS is a form of technology, designed to provide information to clinicians at the time of a decision to improve clinical judgment.^1–4^ In order for a CDSS to be implemented and adopted into clinical practice, it must be considered usable and useful to the end users of the technology.^5^^,^^6^ A systematic review of CDSSs found little evidence that these systems improved clinician diagnostic performance. It was suggested that 1 method to address this issue is to better understand and improve human-computer interaction prior to CDSS implementation.^7^ For this reason, early evaluation of the usability and usefulness of CDSSs is important to increase the likelihood of successful implementation and adoption. However, for CDSSs designed for clinicians treating patients with medical emergencies, few usability studies exist to guide the development process of these technologies.

Usability is defined as a “quality attribute that assesses how easy interfaces are to use”, which has several components: learnability, efficiency, memorability, errors, and satisfaction.^8^ The ISO (International Organisation for Standardisation) Standard 9241-11:2018 defines usability more specifically as “the extent to which a product can be used to achieve specified goals with effectiveness, efficiency, and satisfaction in a specified context of use”.^9^ A recent systematic review showed that almost half of studies also described usefulness as a usability metric.^10^ Usefulness refers to the degree to which using a technology will enhance job performance.^11^

Mobile health (mHealth) refers to applications (apps) which are developed on handheld devices (such as smartphones or tablets) for use in healthcare—either by healthcare professionals, patients, or carers.^12^ The potential benefits of mHealth to healthcare systems include time saving, reduced error rates, and cost savings.^13^^,^^14^ Types of app uses include diagnostics and decision-making, behavior change intervention, digital therapeutics, and disease-related education.^14^ There are numerous apps tailored to specific professions, specialties, patient groups, or clinical situations, including healthcare emergencies.^15^^,^^16^

Some CDSSs have been designed for use in healthcare emergencies. Healthcare emergencies can be defined as any situation where a person requires immediate medical attention in order to preserve life or prevent catastrophic loss of function. There are multiple clinical situations which could be considered healthcare emergencies, and many healthcare professionals who may care for these patients. Examples include problems with the patient’s airway (eg, airway obstruction), breathing (eg, pulmonary embolism), circulation (eg, heart attack or stroke), or multi-system conditions such as injury or burns.^17^^,^^18^ These scenarios are time-critical, requiring timely decision-making and action.

### Study motivation

Design of mobile CDSSs used in healthcare emergencies is important because it must be easy to use, useful, and seamlessly fit into the clinical workflow. The input must be minimal and ideally automatic, while the outputs must be simple, intuitive, and immediately applicable in order to avoid workflow disruption.^19–21^ Usability of CDSSs designed for emergencies is therefore arguably more important than for CDSSs designed for nonemergency (ie, elective) clinical settings.

There are multiple methods of usability testing. Though systematic reviews have been published which address usability methods used for CDSS evaluation,^10^^,^^22–25^ none have focused on mobile CDSSs designed or used in healthcare emergencies. For stakeholders, including academics, clinicians, healthcare managers, and information technologists, who are designing mobile CDSS for use in healthcare emergencies, the methods for testing usability, and associated standards must be understood in this unique context.

## OBJECTIVE

This study answers the question: “What methods are employed to assess the usability of mobile clinical decision support systems designed for clinicians treating patients experiencing medical emergencies?” Our primary aim was to determine the methods of usability evaluation used by researchers of mobile healthcare decision support in clinical emergencies. Our secondary aims were to determine the characteristics of healthcare decision support in emergencies which underwent usability evaluations; and to determine the quantitative and qualitative standards and results achieved, utilizing descriptive quantitative and qualitative evidence synthesis (Supplementary Table S1).

## MATERIALS AND METHODS

This systematic review was conducted according to the Preferred Reporting Items for Systematic reviews and Meta-Analysis (PRISMA) guidelines (Supplementary Table S2),^26^ and it was prospectively registered with the PROSPERO database, ID number CRD42021292014.^27^

### Search strategy

**Figure 1.**
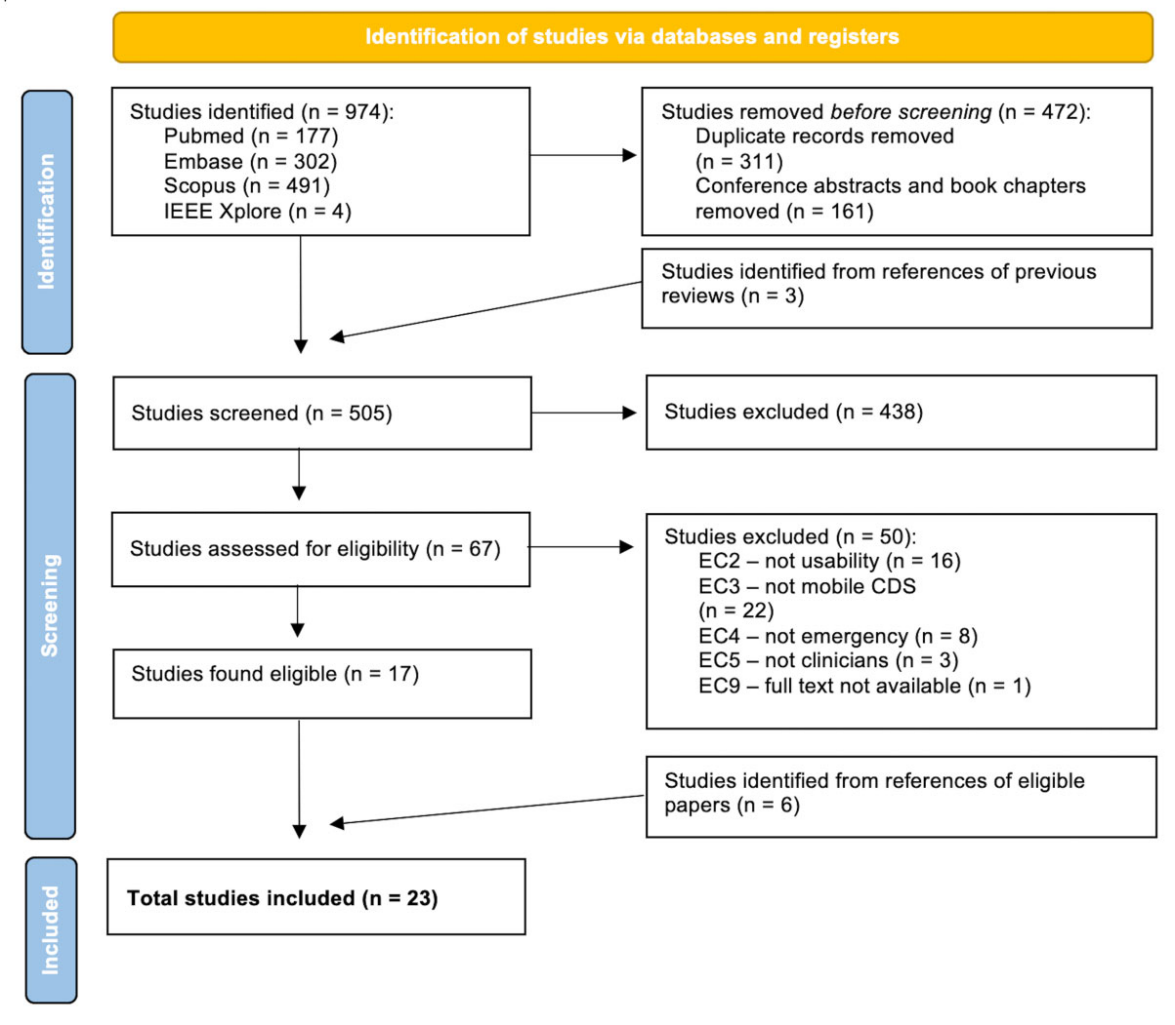
PRISMA flow diagram.

Relevant publications were identified by an electronic search of the Pubmed/Medline, Embase, Scopus, and IEEE Xplore databases using combinations of the following keywords and their synonyms: “usability”, “assessment”, “mobile”, “application”, “decision support”, “healthcare”, and “emergency”. The full search strategy is available in Supplementary Table S3. Searches were limited to Title and Abstract, and English-language only (Supplementary Table S4). The search was performed on December 9, 2021. The search results were uploaded to Endnote X9.3.3 (Clarivate analytics, Philadelphia, PA, USA), in order to identify and delete duplicates, conference abstracts, and book chapters. Two authors (JW and EP) independently screened individual citations against the inclusion criteria using Rayyan software (Rayyan Systems Inc, Cambridge, MA, USA).^28^ Two authors then independently assessed the full text of all identified citations for eligibility. Disagreements were resolved by a third independent reviewer (EK). Reasons for excluding studies were recorded (Figure 1). The reference lists of included articles, as well as excluded systematic reviews, were searched to identify additional publications.

### Eligibility criteria and study designs/settings

Inclusion and exclusion criteria are listed in Table 1. The study eligibility criteria used the PECOS (population, exposure, comparator/control, outcomes, study designs/settings) framework. The *population* was any study testing/evaluating usability using human participants. The *exposure* was any study which tested usability of a healthcare-related mobile application which provided clinical decision support to clinicians. There was no *comparator/control* used. The *outcomes* included studies which provided empirical results from an evaluation of a system’s usability (either quantitative, qualitative, or both). The *setting* was studies which evaluated a CDSS which was designed for use by clinicians in healthcare emergencies.

**Table 1.** Inclusion and exclusion criteria

**Inclusion criteria**	
1	The paper tests/evaluates usability
2	The paper is focused on a healthcare-related technology/application/software/system including mobile, smartphone, tablet, digital, electronic, handheld/portable device, or website
3	The paper provides empirical results (quantitative or qualitative)
4	The system provides decision support/aid/tool, or risk prediction, or prognosis or diagnosis for decision-making
5	The system is designed for use in healthcare emergencies
**Exclusion criteria**	
1	Not written in English
2	Not testing usability, or does not describe the methods adequately
3	Not mobile clinical decision-support
4	Not designed for or tested in clinical emergencies
5	Not targeting clinicians as users
6	Not human participants
7	Not an empirical study (is a theory or review paper)
8	Study protocol only
9	Full text is not available

### Quality of studies assessment

The methodological quality of included studies were assessed using a modified Downs and Black (D&B) checklist by 1 study author (JW).^29^ The D&B checklist was developed to evaluate the quality of both randomized and nonrandomized studies of healthcare interventions on the same scale.^29^ We omitted questions 5, 9, 12, 14, 17, 25, 26 of the 27, because they were deemed not appropriate for assessing the included papers’ methods of usability assessment (Supplementary Table S5).^10^ We did not exclude articles due to poor quality. Quality of Studies (QOS) was classified according to the proportion of modified D&B categories present per paper, as low (<50%), medium (50–74%), and high (≥75%) quality.

### Data extraction

Data were extracted and tabulated in Microsoft Excel (Microsoft, Redmond, WA, USA), according to the study aims (Supplementary Table S1). Demographic data were collected by JW. Two authors (JW and EP) independently extracted data relating to the study aims, using a standardized proforma, which were combined for analysis. Any discrepancies were resolved by consensus. The following data were extracted from each study: Study demographics (citation details, country of study conduct, type of study); Aim (1) method of usability evaluation, including usability definition, metrics and methods used to evaluate usability, number and characteristics of participants, and quantitative and qualitative results reported; Aim (2) characteristics of the CDSS, including type and number of medical specialties targeted, number and type of conditions targeted, CDSS input (number, type, method, and description), CDSS computation (complexity, method, and description), CDSS output (number, type, and description), device used, guideline on which the CDSS is based, stage of CDSS (Development, Feasibility, Evaluation, Implementation),^30^ and CDSS name and description (Supplementary Table S1). Supplemental material was sought if available. Any links in the paper to external information (app website, web calculator, etc.), or articles cited which contain missing information (such as published article describing app development) were sought. Missing or unclear information was discussed between JW and EP, and if uncertainty remained, study authors were contacted. Missing data were not included in quantitative or qualitative analysis for individual study metrics.

### Strategy for data synthesis

Data synthesis was descriptive only for quantitative data addressing the primary and secondary outcomes. Results from individual studies were summarized and reported individually, with no meta-analysis planned or performed.

To describe the qualitative standards and results achieved of assessing usability of CDSSs in medical emergencies, qualitative evidence synthesis methods were used. The PerSPecTIF (perspective, setting, phenomenon of interest, environment, comparison, timing, and findings) question formulation framework was used to define the context and basis for qualitative evidence synthesis (Supplementary Table S6).^31^ Inductive thematic analysis of qualitative results in included studies was undertaken to identify usability-related barriers and facilitators to adoption of mobile CDSS in healthcare emergencies, using a 6-step inductive thematic analysis method: (1) familiarization with the data, (2) generating initial codes, (3) searching for themes, (4) reviewing themes, (5) defining and naming themes, and (6) producing the report/manuscript.^32^ For qualitative evidence synthesis, our research questions were “what were the themes of usability-related barriers to, and facilitators of adoption of mobile CDSS in emergency settings, and what is the relationship between these themes and the method used to assess usability?” Qualitative data were extracted from individual studies and imported into NVIVO software version 12.0 (QSR International, Melbourne, Australia).

## RESULTS

### Study inclusion

The systematic search identified 974 studies. Of 505 unique full-text studies, 67 appeared to meet inclusion criteria from screening, and 23 were included in the analysis after full-text review (Figure 1). For 7 studies, there was disagreement between 2 reviewers after full text review, in which the papers appeared to meet inclusion. A third reviewer (EK) included 4 of these, excluding 3 papers: 1 because it was not usability,^33^ 1 because it was not testing mobile CDSS,^34^ and 1 because it was not a healthcare emergency.^35^ Overall, key reasons for exclusions (*n* = 50) were the paper did not evaluate usability (*n* = 16), did not report mobile clinical decision support (*n* = 22), was not a healthcare emergency (*n* = 8), did not assess clinicians (*n* = 3), or full text was unavailable (*n* = 1) (Figure 1).

### Characteristics of included studies

Twenty studies (87%) were observational, 1 was a randomized controlled trial,^36^ 1 was a proof of concept experiment,^37^ and 1 was a pilot nonrandomized controlled study (Table 2; Supplementary Table S7).^38^ All included studies were published between 2003 and 2021. The majority of studies (*n* = 13; 57%) were published between 2017 and 2021, with 8 (35%) studies published between 2012 and 2016, and 2 (9%) published between 2002 and 2011. The geographical distribution of studies, by participant location, included 8 in Europe (35%), 6 in North America (26%), 5 in Africa (22%), 3 in Asia (13%), and 1 in South America (4%). The most common method used to assess usability was a questionnaire (*n* = 20; 87%), followed by user testing (*n* = 17; 74%), interviews (*n* = 6; 26%), and heuristic evaluations (*n* = 3; 13%). Combinations of these methodologies were also used, with a quarter (*n* = 6; 26%) of studies using 1 method, half (*n* = 11; 48%) using 2 methods, and a quarter (*n* = 6; 26%) using 3 methods. Quantitative methods were used in 10 (43%) studies, qualitative methods in 1 (4%) study, and both quantitative and qualitative methods were used in 12 (52%) studies.

**Table 2.** Characteristics of included studies

Year	First author and reference	Country^a^	Study design	Methods^b^	Validated methods	Participants	Conditions	Device	Name of system	Guideline on which CDSS is based	Stage(s) of CDSS^c^
2015	Barnes^39^	UK	Observational, comparative (app vs paper)	Q, U	NA	Medical students	Burns	Mobile (smartphone, tablet)	Mersey Burns App	Parkland formula for burns	Evaluation and implementation
2003	Chang^40^	Taiwan	Observational, comparative (PDS vs terminal)	Q	TAM^6^	Emergency medical staff	Multiple: allergy, hypertension, diabetes, trauma, nontrauma	Mobile (PDA)	NA	NA	Development and feasibility
2004	Chang^41^	Taiwan	Observational	Q	TAM^6^	Emergency medical staff	Multiple: mass gathering-related, including trauma and infectious disease	Mobile (PDA)	NA	NA	Feasibility
2019	Clebone^42^	USA	Observational	Q, U	SUS^43^	Anesthetists	Multiple: airway, nonairway	Mobile (smartphone)	Pedi Crisis 2.0 App	Society for Pediatric Anesthesia 26 Pediatric Crisis checklists	Development and feasibility
2020	Corazza^38^	Italy	Pilot nonrandomized controlled	Q, U, I	UEQ,^44^ NASA-TLX^45^	Pediatric clinicians	Pediatric cardiac arrest	Mobile (tablet)	PediARREST App	American Heart Association Pediatric Advanced Life Support 2015	Development and feasibility
2021	Ellington^46^	Uganda	Observational	U, I	NA	Pediatric clinicians	Pediatric acute lower respiratory Illness	Mobile (smartphone)	ALRITE	WHO Integrated Management of Childhood Illnesses—Acute Lower Respiratory Illnesses guidelines	Development and feasibility
2015	Frandes^47^	Romania	Observational	Q	NA	Physicians and nurses	Diabetic ketoacidosis (DKA)	Mobile (smartphone, tablet)	mDKA	Medical standards for diabetes care	Development and feasibility
2015	Ginsburg^48^	Ghana	Observational	Q, U, I	SUS^43^	Mixed medical staff	Childhood pneumonia	Mobile (tablet)	mPneumonia	WHO Integrated Management of Childhood Illnesses guidelines	Development and feasibility
2016	Ginsburg^49^^,^^50^	Ghana	Observational	Q, U, I	SUS^43^	Mixed medical staff	Childhood pneumonia	Mobile (tablet)	mPneumonia	WHO Integrated Management of Childhood Illnesses guidelines	Feasibility
2017	Khodambashi^51^	Norway	Observational	Q, U, I	SUS^43^	Emergency medical staff	Mental illness (suicidal or violent)	Mobile (smartphone, tablet)	NA	Norwegian laws related to forensic psychiatry	Development and feasibility
2018	Klingberg^52^	South Africa	Observational	Q, U, I	Health-ITUES^53^	Emergency medical staff	Burns	Mobile (smartphone)	Vula App	Burns size calculation and Parkland formula	Evaluation and implementation
2020	Klingberg^54^	South Africa	Observational	Q	TAM,^6^ IDT,^55^ and TPB^56^	Physicians and nurses	Burns	Mobile (smartphone)	Vula App	Burns size calculation and Parkland formula	Feasibility
2014	O'Sullivan^57^	Canada	Observational	Q	NA	Pediatric clinicians	Asthma exacerbations	Mobile (tablet); Desktop (web app)	MET3-AE	Bayes prediction of asthma exacerbation severity within 2h of nursing triage	Development and feasibility
2018	Paradis^58^	Canada	Observational	Q, U	TRI^59^	Physicians and nurses	Multiple: knee, ankle, and neck injuries	Mobile (smartphone, tablet)	Ottawa Rules App	The Ottawa Rules	Feasibility and evaluation
2020	Quan^60^	Canada	Observational	Q, U	TRI^59^	Physicians and nurses	Multiple: knee, ankle, neck, and head injuries	Mobile (smartphone, tablet)	Ottawa Rules App 3.0.2	The Ottawa Rules	Feasibility and evaluation
2020	Rodriguez^61^	Colombia	Observational	Q	mERA,^62^ iSYScore index,^63^^MARS,^^64^ and uMARS^65^	General practitioners	Multiple: acute febrile syndromes	Mobile (smartphone)	FeverDx	Colombian Ministry of Health’s clinical practice guidelines for diagnosis and management of arboviruses	Development and feasibility
2019	Schild^66^	Germany	Observational	Q, U	SUS^43^	Anesthetists	Multiple: anesthetic emergencies	Mobile (tablet); Desktop (web app)	NA	German Cognitive Aid Working Group	Development and feasibility
2016	Schoemans^37^	Belgium	Proof of Concept Experimental	Q, U	TAM^6^ and PSSUQ^67^	Physicians, nurses, data managers, and students	Graft versus host disease (GVHD)	Desktop (web app)	eGVHD App	Acute (Glucksberg and IBMTR scores) and chronic (NIH criteria) GVHD	Development and feasibility
2018	Schoemans^36^	Belgium	Randomized Controlled Trial	Q, U	TAM^6^ and PSSUQ^67^	Physicians, data managers, other	Graft versus host disease (GVHD)	Mobile (smartphone, tablet); Desktop (web app)	eGVHD App	Acute (Glucksberg and IBMTR scores) and chronic (NIH criteria) GVHD	Evaluation
2018	Schoemans^68^	France	Observational, comparative (app vs self-assessment)	Q, U	NA	Physicians, nurses, data managers, other	Graft versus host disease (GVHD)	Mobile (smartphone, tablet, laptop)	NA	Acute (Glucksberg and IBMTR scores) and chronic (NIH criteria) GVHD	Feasibility
2020	Sutham^69^	Thailand	Observational, comparative (app vs handbook vs experienced)	U, H	Nielsen’s Heuristics^70^	Emergency medical staff	Multiple: trauma, nontrauma	Mobile (smartphone)	Triagist App	National Institute for Emergency Medicine of Thailand Criteria-Based Dispatch	Development and feasibility
2015	Yadav^71^	USA	Observational	U, H	Nielsen’s Heuristics^70^	Pediatric clinicians, usability engineers	Pediatric head injuries	Desktop (web app)	NA	Pediatric Emergency Care Applied Research Network clinical decision rule for head CT	Development and feasibility
2013	Yuan^72^	USA	Observational	Q, U, H	NASA TLX,^45^ Nielsen’s Heuristics^70^	Nurses, information scientist	Multiple: heart attack, pleurisy, reflux/indigestion, pneumothorax, myocardial infarction	Mobile (tablet)	NA	NA	Development and feasibility

a Country of study conduct.

b Q, U, I, H are questionnaire, user-testing, interview, and heuristic evaluation studies, respectively;.

c Stage(s) of CDSS (Development, Feasibility, Evaluation or Implementation) are based on MRC/NIHR framework for developing and evaluating complex interventions.^30^

NA: not applicable; TAM: technology acceptance model; SUS: system usability scale; UEQ: user experience questionnaire; NASA TLX: National Aeronautics and Space Administration task load index; Health-ITUES: health information technology usability evaluation scale; IDT: innovation diffusion theory; TPB: theory of planned behavior; mERA: mobile health evidence reporting and assessment checklist; MARS: mobile application rating scale; uMARS: user version of the mobile application rating scale; PSSUQ: poststudy system usability questionnaire; TRI: technology readiness index.

Studies used a number of validated tools to assess usability: The system usability scale (SUS^43^), and the technology acceptance model (TAM^6^) were each included in 5 (22%) studies, Nielsen’s Heuristics^70^ in 3 (13%) studies, NASA Task Load Index (TLX) in 2 (9%) studies, technology readiness index (TRI) in 2 (9%) studies, the poststudy system usability questionnaire (PSSUQ) in 2 (9%) of studies, and 8 other validated methods were used in 1 included study each (Table 2). Five (22%) studies used no validated method. All studies included clinician participants, while 3 studies also included data managers,^36^^,^^37^^,^^68^ 1 study included usability engineers,^71^ and 1 study had information scientists as participants.^72^

### Characteristics of mobile CDSSs in healthcare emergencies

The targeted emergency conditions included multiple conditions in 9 (39%) studies,^40–42^^,^^58^^,^^60^^,^^61^^,^^66^^,^^69^^,^^72^ burns in 3 studies (13%),^39^^,^^52^^,^^54^ graft versus host disease in 3 studies (13%),^36^^,^^37^^,^^68^ pediatric respiratory illness in 3 studies (13%),^46^^,^^48^^,^^50^ and 1 study addressing each of: pediatric cardiac arrest,^38^ diabetic ketoacidosis,^47^ mental illness (suicidal or violent),^51^ asthma,^57^ and pediatric head injuries (Supplementary Table S7).^71^ Nine studies evaluated mobile CDSS designed for multiple device types,^36^^,^^39^^,^^47^^,^^51^^,^^57^^,^^58^^,^^60^^,^^66^^,^^68^ 6 for smartphones,^42^^,^^46^^,^^52^^,^^54^^,^^61^^,^^69^ 4 for tablets,^38^^,^^48^^,^^50^^,^^72^ 2 for desktop web apps,^37^^,^^71^ and 2 for personal digital assistants.^40^^,^^41^ Nearly, all CDSSs (*n* = 20; 87%) were based on a guideline, and most were in development (*n* = 14; 61%) or feasibility (*n* = 20; 87%) stages, while a minority were in evaluation (*n* = 5; 22%) or implementation (*n* = 2; 9%) stages. The majority (*n* = 18; 78%) of CDSSs required manual checkbox/radio button inputs, with a minority (*n* = 2; 9%) incorporating a form of automatic input (Supplementary Table S7). Nearly, all (*n* = 22; 96%) had text output, while nearly half (*n* = 10; 43%) had numerical input, and few (*n* = 2; 9%) had image or video (*n* = 1; 4%) input. A majority (*n* = 18; 78%) of CDSSs provided a clinical recommendation, a quarter (*n* = 6; 26%) a specific treatment, and a quarter (*n* = 6; 26%) a score, risk level, likelihood of diagnosis (Supplementary Tables S7 and S8). Over half (*n* = 13; 57%) of studies had descriptions of the number of CDSS inputs: Of these, there were a median of 50 inputs (interquartile range [IQR] 11–78) (Supplementary Table S8). Twenty (87%) studies had descriptions or figures outlining the number of CDSS output; of these, there were a median of 2 outputs (IQR 1–3) (Supplementary Table S8).

### Quality of studies

**Figure 2.**
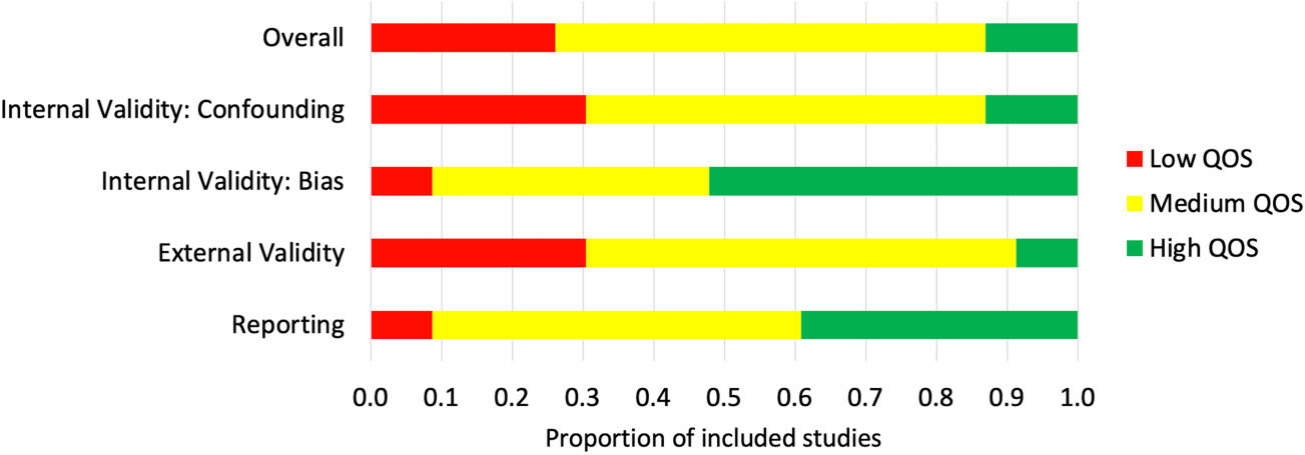
Quality of studies (QOS) summary: the proportion of included studies which scored low, medium or high, overall and for each QOS subcategory.

**Figure 3.**
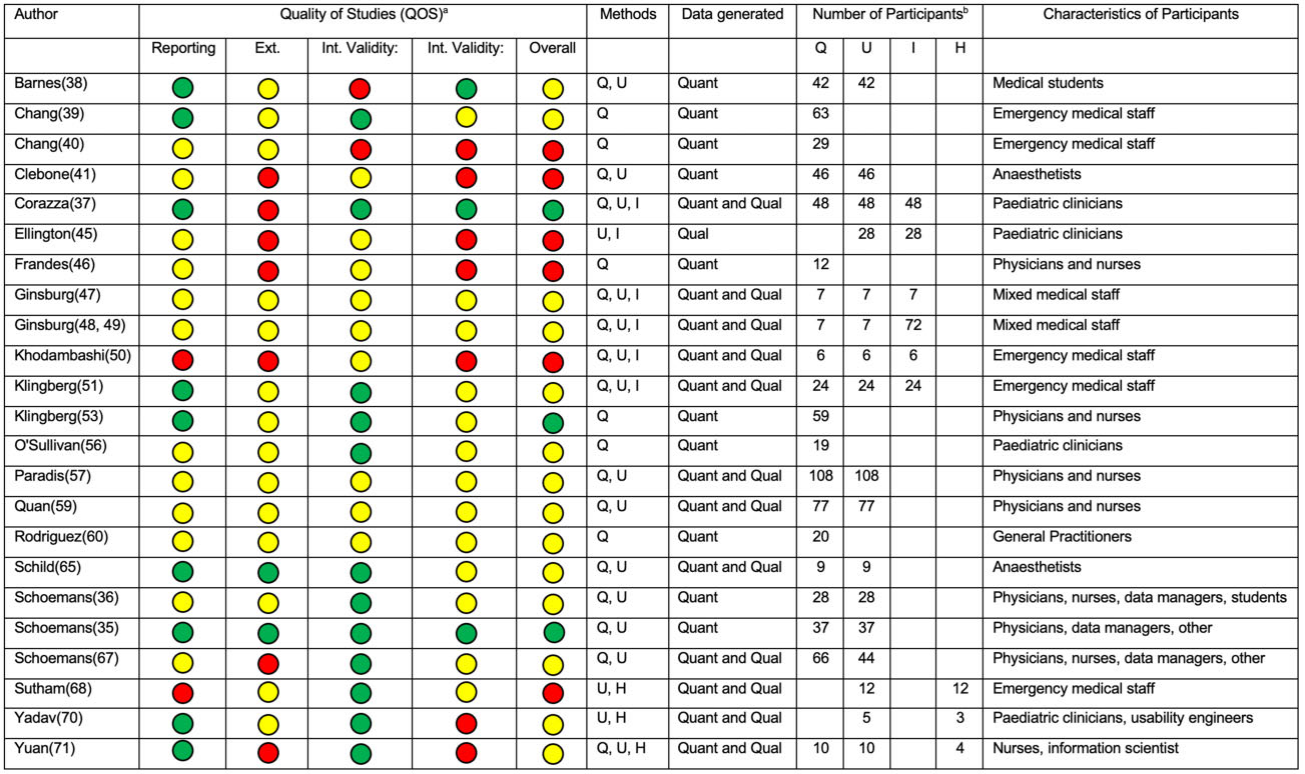
Quality of studies (QOS) summary and individual study characteristics. ^a^Green: high QOS; yellow: moderate QOS; red: low QOS. ^b^Q, U, I, H are questionnaire, user-testing, interview, and heuristic evaluation studies, respectively. Int: internal; Ext: external; Quant: quantitative; Qual: qualitative.

Results for the modified Downs and Black (D&B) quality assessment of included studies (QOS) showed that overall, only 3 studies (13%) had high QOS, 14 (61%) had medium QOS, and 6 (26%) had low QOS (Figure 2). Studies which employed more methods to evaluate usability did not have a substantial difference in risk of bias (Figure 3). There was, however, lower risk of bias overall in studies which used mixed methods (both qualitative and quantitative), rather than only quantitative or only qualitative methods of usability evaluation (Figure 3). A median of 29 (IQR 12–51) participants were recruited for questionnaire-based studies, 28 (IQR 9–44) participants for user trials, 26 (IQR 11–43) participants for interview-based studies, and 4 (IQR 4–8) participants for heuristics studies.

### Definition of usability in included studies

Of the 23 included studies, 13 (57%) did not define usability. Of the 10 which provided a definition, 3 (30%) used the definition provided by the ISO (ISO 9241-11),^9^ which is the “extent to which a system, product or service can be used by specified users to achieve specified goals with effectiveness, efficiency and satisfaction in a specified context of use”.^51^^,^^52^^,^^57^ Two (20%) defined usability as “the design factors that affect the user experience of operating the application’s device and navigating the application for its intended purpose”.^46^^,^^50^ Other definitions of usability included:

- Differentiating “content usability” (data completeness and reassurance of medical needs), from “efficiency improvement” (quicker and easier evaluation), and “overall usefulness of systems”^41^
- “ease of use, confidence in input, preference in an emergency setting, speed, accuracy, ease of calculation, and ease of shading”^39^
- “efficiency, perspicuity, dependability”^38^
- “functionality, convenience, triage accuracy, and accessibility.”^69^

### Usability evaluation metrics used

**Figure 4.**
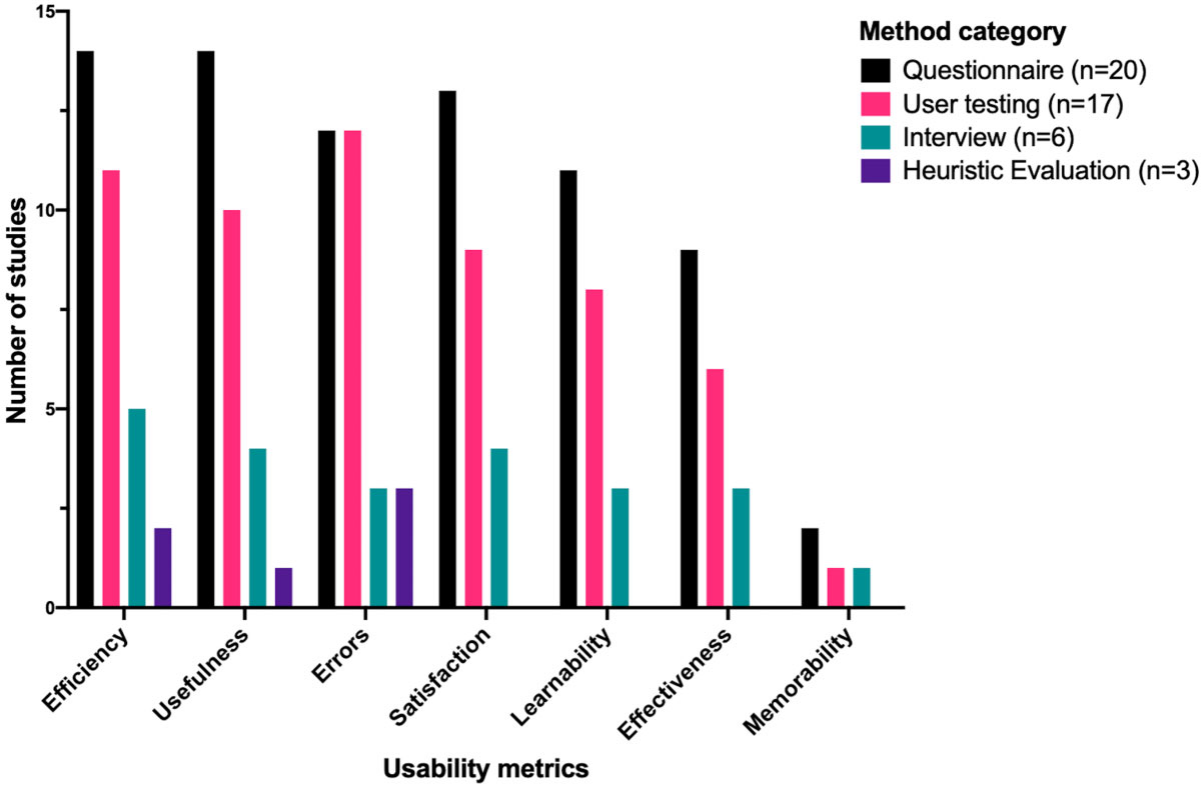
Usability metrics evaluation in the included studies, presented as the number of metrics use in studies using each method. Ordered from most-commonly used on the left, to least commonly used on the right.

Though not all studies defined usability explicitly, all studies reported how usability was evaluated. The most frequent evaluation metrics were Efficiency and Usefulness, measured in 15 (65%) studies. User Errors were measured in 14 (61%), Satisfaction in 13 (57%), Learnability in 11 (48%), Effectiveness in 9 (39%), and Memorability in 2 (9%) studies. The frequency of usability evaluation metrics was similar between studies utilizing questionnaire, user testing, and interview methods, though studies using heuristics only measured Usefulness, Efficiency, and user Errors (Figure 4).

### Description of quantitative results

Descriptive quantitative results from included studies are summarized in Supplementary Table S9. The 5 studies which used SUS as a method all achieved acceptable usability scores (>67). The 5 studies which used TAM as a method achieved mixed results, with 1 study demonstrating worse usability than the existing system,^40^ and another study having different usability depending on user group (physicians vs nurses).^41^ Both studies which used NASA TLX to measure mental effort found it was acceptably low, with 1 study stating that perceived workload was comparable whether the app was used or not.^38^ Of the 2 studies which employed the TRI, 1 found no difference based on demographics, and 1 found that younger users were more ready for the technology.^60^ Of the 3 studies which employed Nielsen’s Heuristics, 2 identified usability issues in each of the 10 design heuristics categories.^71^^,^^72^

### Qualitative results synthesis

Themes of usability-related barriers to adoption included: external issues, hardware issues, input problems, output problems, poor software navigation, poor user interface design, user barrier, and user emotion or experience (Table 3). A higher proportion of codes (of barriers and facilitators to adoption) were generated by interviews and heuristics evaluation methods, than questionnaire or user testing methods (Table 3). Themes of usability-related facilitators of adoption included: automaticity, user interface design, efficiency, feasibility, learnability, patient benefit, trustworthiness, ease of use, usefulness, and user experience (Table 4). A more complete identification of themes (of barriers and facilitators to adoption) occurred when included studies used interviews and heuristic evaluation, compared to user testing or questionnaire (Table 4).

**Table 3.** Qualitative evidence synthesis of included studies (*n* = 13/23): usability-related themes and codes of barriers to adoption, by usability method category

Themes	Q	U	I	H	Codes	Q	U	I	H
External issues	0	0	3	1	External issues	0	0	3	1
Hardware issues	0	3	5	1	Hardware issues	0	3	5	1
Input problems	4	6	37	24	Difficult tasks	0	0	2	2
					Inaccurate results	0	1	3	0
					Instructions unclear	1	1	11	9
					Mismatch with reality	0	1	2	2
					Not automated	1	0	0	1
					Not efficient	1	3	7	1
					Not enough information	1	0	2	4
					Not incorporating standard practices	0	0	1	2
					Not intuitive	0	0	9	3
Output problems	0	1	10	10	Interrupting workflow	0	1	2	1
					Minimizes group situational awareness	0	0	0	1
					Not clinically useful	0	0	2	3
					Not updating user	0	0	2	5
					Recommendations unclear	0	0	4	0
Poor software navigation	1	7	8	3	Poor software navigation	1	7	8	3
Poor user interface design	4	5	16	15	Poor user interface design	3	5	14	11
					Information overload	1	0	1	1
					Poor formatting	0	0	1	3
User barrier	2	10	29	6	Impact on other patients	0	0	1	0
					Lack of familiarity	0	4	8	1
					Medico-legal concern	0	1	0	0
					Need for training	0	1	5	0
					Not used as intended	1	0	1	1
					Patient not willing	0	0	3	0
					User mistakes	1	4	11	4
User emotion or experience	0	0	14	3	Fear to use	0	0	2	0
					Frustration when using	0	0	3	1
					Hesitancy towards CDSS	0	0	1	1
					Not understanding instructions	0	0	5	1
					Purpose needs explaining	0	0	2	0
					Uncomfortable when using	0	0	1	0
Total themes identified	4	6	8	8	Total codes identified	11	32	122	63
Themes missed	4	2	0	0	Codes missed	24	21	3	9
Proportion identified (*n* = 8)	50%	75%	100%	100%	Proportion identified (*n* = 33)	27%	36%	91%	73%

Q: questionnaire; U: user testing; I: interview; H: heuristic evaluation studies.

**Table 4.** Qualitative evidence synthesis of included studies (*n* = 13/23): usability-related themes and codes of facilitators of adoption, by usability method category

Themes	Q	U	I	H	Codes	Q	U	I	H
Automaticity	0	0	6	5	Automatic functioning	0	0	6	5
User interface design	5	2	13	7	Ability to correct mistake error	0	0	0	2
					Clear design	1	0	1	2
					Few problems	2	1	1	1
					Good design	2	1	3	1
					Good internal (app) flow	0	0	1	0
					Simple design	0	0	4	0
					Familiarity with technology	0	0	2	1
					Size and shape of device	0	0	1	0
Efficiency	1	2	7	1	Time efficiency	1	2	7	1
Feasibility	0	0	4	3	Feasible to implement	0	0	2	0
					Minimally disruptive to work flow	0	0	2	3
Learnability	0	2	2	0	Learnability and intuitiveness	0	2	2	0
Patient benefit	0	0	2	0	Patient benefit including noninvasive	0	0	2	0
Trustworthiness	1	1	15	5	Improves safety	0	0	3	2
					Accuracy	0	1	7	1
					Improves trust	0	0	2	2
					Multiple types of people approve	0	0	1	0
					Thoroughness systematic	1	0	2	0
Ease of use	5	0	7	0	Comforting	1	0	0	0
					Convenience	1	0	0	0
					Easy to use	3	0	7	0
Usefulness	3	0	28	8	Adds knowledge	0	0	2	1
					Help diagnosis	0	0	7	2
					Helpful for communication	0	0	2	1
					Helpful for inexperienced clinicians	2	0	0	1
					Helpful for work	0	0	6	0
					Important information prominent to user	0	0	0	3
					Improves assessment	0	0	3	0
					Improves patient management	0	0	2	0
					Leads to increased demand for services	0	0	1	0
					Reduces paperwork	0	0	1	0
					Useful	1	0	3	0
					Useful in other contexts	0	0	1	0
User experience	0	0	13	0	Novelty of technology	0	0	3	0
					Practice and instruction	0	0	4	0
					Good user experience	0	0	1	0
					Preference compared to current method	0	0	2	0
					Word of mouth positive	0	0	1	0
					Would use again	0	0	2	0
Total themes identified	5	4	10	6	Total codes identified	15	7	97	29
Themes missed	5	6	0	4	Codes missed	30	35	5	24
Proportion identified (*n* = 8)	50%	40%	100%	60%	Proportion identified (*n* = 40)	25%	13%	88%	40%

Q: questionnaire; U: user testing; I: interview; H: heuristic evaluation studies.

## DISCUSSION

The standardized framework for defining usability (ISO) was established in 1998, and updated in 2018 (ISO 9241-11:2018).^9^ Despite this, the majority of included papers in this review demonstrated deviation in the definition of usability used. Importantly, this standard does not describe specific methods of design, development, or evaluation of usability. Nevertheless, differing definitions of usability likely contributed to the evidence generated from this systematic review, which revealed that a wide range of metrics and methods are used to assess usability of mobile CDSSs. Researchers favored evaluation metrics, including efficiency, user errors, usefulness, and satisfaction over measures such as effectiveness, learnability, and memorability. Qualitative evidence synthesis including thematic analysis identified that more codes and themes were generated from studies utilizing interview and heuristic evaluation, than studies which employed user testing or questionnaires to assess usability of CDSSs. Synthesis of quantitative results was not attempted, due to the multiple different methods used (validated and nonvalidated) to measure usability quantitatively across included studies.

### Implications

There are 5 main implications of this study. Firstly, the study reveals that a plethora of approaches are evident, which suggests that comparison of usability metrics between different CDSS is inherently difficult and could contribute to confusion and misunderstanding when attempting to understand the value of these tools to practitioners, patients, and health systems. The lack of consistency in evaluating the usability of CDSS is a material problem for the field. In particular, the quantitative approaches used by included studies were so diverse that no meaningful data synthesis could be made. There is a dire need for a standard approach to quantitative analysis on the usability of CDSS. There are multiple validated methodologies in current use.^73^ The best solution likely involves a combination or amalgamation of commonly used methodologies, focusing on those with few items and high reliability.^73^

Secondly, nearly half of included studies evaluated usability using a purely quantitative approach, even though a mixed methods approach may reduce bias.^10^ A mixed methods approach might elicit more complete and useful information when evaluating the usability of CDSS.^10^ However, like quantitative approaches, a plethora of methodological approaches to qualitative analysis exist for the evaluation of usability of CDSS, which makes between-study comparison challenging.^74^ Identifying consistent and shared themes across studies would be more achievable if description and approach of qualitative methodology were explicitly stated.^74^

Thirdly, many CDSSs were designed in ways which hampers their usability. A universal problem with the design of CDSS for mobile use is any reliance on user input, which may be an important fatal flaw for healthcare emergencies. Though studies evaluated mobile CDSSs which were designed for different conditions in multiple emergency settings, most required information to be input manually. Manual information input is a known barrier to usability, and is likely to be particularly burdensome to the end user during clinical emergencies.^19–21^ This study has identified that only a minority of included studies demonstrated any form of automatic data entry system for mobile CDSS, with most utilizing manual checkbox inputs. Automation of CDSS was associated with improved clinical outcome.^75^ Ideally, CDSSs input data automatically in real-time, reducing disruption to clinician workflow, and allowing timely CDSS output.^76^^,^^77^ Physicians make better decisions when they do not have to input the information first, but only integrate available information.^78^ Therefore, automation of data entry should be a focus for future CDSS if they are going to improve their likelihood of implementation and use in emergency settings.

Fourthly, we found divergence with regard to output, with the majority of tools offering a recommendation or specific treatment to clinicians, and a minority providing risk information. The benefits of CDSSs which provide clear recommendations is that they may be easier for clinicians to action than risk information, and therefore increase uptake of CDSSs.^77^ One study demonstrated that CDSSs which provided a recommendation rather than simply an assessment improved clinical outcome.^75^ However, some treatment decisions may be based on factors which cannot be accounted for by the CDSS. Thus, by providing a recommendation, the CDSS is in danger of “overstepping” its bounds, into the realm of decision-making instead of decision support. This is a contentious area, which may also have medico-legal implications if patients come to harm after a clinician provides treatment based on an inaccurate or inappropriate CDSS recommendation. These medico-legal issues become more pertinent for recommendations which are more directive,^2^^,^^3^^,^^79^^,^^80^ though remains a topic of keen interest and debate.^3^^,^^81^

Fifthly, studies which have evaluated CDSSs designed for nonemergency settings, rather than healthcare emergencies, used similar usability methods but different usability metrics. Usability methods were similar between studies included in a recent systematic review (primarily nonemergency settings), and studies included in our review (emergency settings), including questionnaires (78% in nonemergency settings vs 87% in emergency settings), user testing (86% vs 74%), interviews (20% vs 26%), and heuristics evaluations (14% vs 13%).^10^ Conversely, the proportion of studies evaluating usability metrics differed depending on setting, including usefulness (39% in nonemergency setting vs 65% in emergency settings), user errors (31% vs 61%), learnability (24% vs 48%), and memorability (2% vs 9%). More studies evaluated satisfaction (75% vs 57%) and effectiveness (61% vs 39%) of CDSS in the nonemergency setting compared to the emergency setting, and a similar proportion evaluated efficiency (63% vs 65%).^10^ That researchers evaluated different metrics may denote differences in end-user priorities based on setting. For a CDSS to be used in emergencies, it must be useful compared to other competing priorities,^6^ have a low propensity for user errors given the user’s cognitive load,^82^ and be easy to use, learn, and remember.^6^^,^^82^ Automatic data entry may reduce user errors,^75–78^ and more directive recommendations may be easier to apply cognitively than risk percentages alone.^75^^,^^77^ In clinical emergencies, clinicians are focused on the patient’s immediate care needs. Consequently, using a CDSS in this setting may be more prone to user error than in the elective setting. While measuring user errors in the evaluation stage is important, ensuring CDSS design and development follows best principles of user interface design is key to reducing the propensity for user errors in the first place. However, the heterogeneity of usability metrics evaluated in studies provides an impetus for a more standardized approach so that studies can be meaningfully compared, regardless of setting.

Similar literature exists which corroborates our findings regarding user errors, effectiveness, and efficiency. A user error is defined as either a slip (unintended action with correct goal; ie, misspelling an email address), or a mistake (intended action with incorrect goal; ie, clicking on an un-clickable heading), and can highlight interface problems.^83^ Effectiveness (or “success”) is defined as the number of successfully completed tasks or the percentage of correct responses; while efficiency is the time taken, or number of clicks required, to complete a task.^10^ In the same systematic review as above, focusing on usability metrics within usability evaluation studies, 31% of studies measured user errors.^10^ These included 23 different user error measurement techniques, while the number of user errors or percentage of user errors were most frequently reported. Conversely, effectiveness was measured in 61% of studies, and efficiency measured in 63% of studies. The study concluded that there are multiple methods to evaluate usability, each with benefits and deficiencies. To mitigate these and provide the most complete usability evaluation, a combination of multiple methods is advised.

### Limitations

There are several limitations to this review. First, while we provided a synthesis of the qualitative results provided by included studies, it was impossible to synthesize the quantitative data in a meaningful way due to their heterogeneity. Further, while the qualitative analysis was conducted using a robust method,^32^ and framework,^31^ synthesizing qualitative results from studies with heterogeneous designs may produce unreliable results. Second, while it is recognized that a description which weighted usability methods to determine which methods are better would be desirable, this was not our aim. Rather, we provided a descriptive summary of quantitative outcomes achieved, and synthesis of qualitative results, to highlight the relative benefits of different methodological approaches to usability evaluation, with regards to the ability of each method to identify barriers and facilitators to CDSS adoption. Structural differences in study methodology will have impacted results, such that questionnaires and user testing studies often did not allow open responses to elicit additional user input, resulting in comparatively more qualitative information from interview and heuristic evaluation studies. Third, the narrow search criteria did not account for recent technical developments, including the rapid pace of CDSS utilizing machine learning and artificial intelligence. Accordingly, though the review protocol included a goal to determine trends over time in healthcare decision support in emergencies, including how statistical or computational complexity and devices have changed over time, our search yielded studies which demonstrated little variation in either of these parameters. This question may be best answered by a scoping review or narrative literature review. The authors considered Google Scholar as a search engine in order to broaden the review’s inclusion, but decided against it due to evidence reporting its imprecision as a systematic search engine.^84^^,^^85^ Fourth, studies were not excluded based on assessed quality, and 5 did not use validated methods to assess usability. However, the authors preferred a “real-world” evaluation of available literature. Fifth, this paper evaluates methods and metrics of usability of CDSSs which were largely in development and feasibility stages, with only a small minority in the evaluation or implementation stages. Therefore, results may be less generalizable to studies which evaluate usability of CDSS in later stages, including implementation and adoption.

## CONCLUSION

Usability evaluation of mobile CDSS in medical emergencies is heterogeneous. Studies evaluated multiple aspects of usability in a variety of study designs. More questionnaires and user testing studies were conducted than interviews and heuristics evaluations. However, interviews and heuristic evaluations identified a greater proportion of the usability issues than did questionnaire and user testing studies. The findings have future research implications on both the design of CDSSs and the evaluation of their usability. Developers should acknowledge that automatic data input into a CDSS may improve its usability, and that outputs which provide a clinical recommendation may be controversial. When planning CDSS usability evaluation studies, developers should consider multiple methods to comprehensively evaluate usability, including qualitative and quantitative approaches. Researchers should apply a more standardized approach to usability evaluation in mobile CDSS while considering the context and workflow.

## Supplementary Material

ooad051_Supplementary_Data

## Data Availability

Template data collection forms, data extracted from included studies, data used for all analyses, and qualitative synthesis are all available upon request from the authors.

## Notes

Protocol Registration on December 16, 2021: Jared M. Wohlgemut, Erhan Pisirir. Usability of mobile clinical decision support systems designed for clinicians treating patients experiencing medical emergencies: a systematic review. PROSPERO 2021 CRD42021292014. Available from: https://www.crd.york.ac.uk/prospero/display_record.php?ID=CRD42021292014.

## References

[1] Berner ES. Clinical Decision Support Systems: State of the Art. AHRQ Publication No. 09-0069-EF [Database]; 2009.

[2] Lyman JA , Cohn WF, Bloomrosen M, et al Clinical decision support: progress and opportunities. J Am Med Inform Assoc 2010; 17 (5): 487–92. doi: 10.1136/jamia.2010.005561. 20819850 PMC2995690

[3] Sutton RT , Pincock D, Baumgart DC, et al An overview of clinical decision support systems: benefits, risks, and strategies for success. NPJ Digit Med 2020; 3: 17. doi: 10.1038/s41746-020-0221-y. 32047862 PMC7005290

[4] Wyatt JC. Decision support systems. J R Soc Med 2000; 93 (12): 629–33. 11193060 10.1177/014107680009301206 PMC1298167

[5] Horsky J , Schiff GD, Johnston D, et al Interface design principles for usable decision support: a targeted review of best practices for clinical prescribing interventions. J Biomed Inform 2012; 45 (6): 1202–16. doi: 10.1016/j.jbi.2012.09.002. 22995208

[6] Davis FD. Perceived usefulness, perceived ease of use, and user acceptance of information technology. MIS Q 1989; 13 (3): 319–40.

[7] Vasey B , Ursprung S, Beddoe B, et al Association of clinician diagnostic performance with machine learning-based decision support systems: a systematic review. JAMA Netw Open 2021; 4 (3): e211276. doi: 10.1001/jamanetworkopen.2021.1276. 33704476 PMC7953308

[8] Nielsen J. Usability 101: Introduction to Usability. Secondary Usability 101: Introduction to Usability; 2012. https://www.nngroup.com/articles/usability-101-introduction-to-usability/. Accessed July 6, 2023.

[9] International Organisation for Standardisation. ISO 9241-11:2018 Ergonomics of Human-System Interaction. Part 11: Usability: Definitions and Concepts. Geneva, Switzerland: ISO; 2018.

[10] Wronikowska MW , Malycha J, Morgan LJ, et al Systematic review of applied usability metrics within usability evaluation methods for hospital electronic healthcare record systems: metrics and evaluation methods for eHealth systems. J Eval Clin Pract 2021; 27 (6): 1403–16. doi: 10.1111/jep.13582. 33982356 PMC9438452

[11] Venkatesh V , Bala H. Technology acceptance model 3 and a research agenda on interventions. Decision Sci 2008; 39 (2): 273–315.

[12] Thomairy NA , Mummaneni M, Alsalamah S, Moussa N, Coustasse A. Use of smartphones in hospitals. Health Care Manag 2015; 34 (4): 297–307. 10.1097/HCM.0000000000000080 26506291

[13] Messner E-M , Probst T, O’Rourke T. mHealth applications: potentials, limitations, current quality and future directions In: Baumeister H, Montag C, eds. Digital Phenotyping and Mobile Sensing: New Developments in Psychoinformatics. Cham: Springer International Publishing; 2019: 235–48.

[14] Rowland SP , Fitzgerald JE, Holme T, et al What is the clinical value of mHealth for patients? NPJ Digit Med 2020; 3: 4. doi: 10.1038/s41746-019-0206-x. 31970289 PMC6957674

[15] Plaza Roncero A , Marques G, Sainz-De-Abajo B, et al Mobile health apps for medical emergencies: systematic review. JMIR Mhealth Uhealth 2020; 8 (12): e18513. doi: 10.2196/18513. 33306037 PMC7762680

[16] Montano IH , de la Torre Diez I, Lopez-Izquierdo R, et al Mobile triage applications: a systematic review in literature and play store. J Med Syst 2021; 45 (9): 86. doi: 10.1007/s10916-021-01763-2. 34387773 PMC8361243

[17] Soar J , Deakin CD, Nolan JP, et al Adult advanced life support guidelines. Secondary Adult advanced life support guidelines; 2021. https://www.resus.org.uk/library/2021-resuscitation-guidelines/adult-advanced-life-support-guidelines.

[18] American College of Surgeons Committee on Trauma. Advanced Trauma Life Support: tenth Edition. 10th ed. Chicago, IL: American College of Surgeons; 2018.

[19] Bates DW , Kuperman GJ, Wang S, et al Ten commandments for effective clinical decision support: making the practice of evidence-based medicine a reality. J Am Med Inform Assoc 2003; 10 (6): 523–30. doi: 10.1197/jamia.M1370. 12925543 PMC264429

[20] Bashiri A , Alizadeh Savareh B, Ghazisaeedi M. Promotion of prehospital emergency care through clinical decision support systems: opportunities and challenges. Clin Exp Emerg Med 2019; 6 (4): 288–96. doi: 10.15441/ceem.18.032. 31910499 PMC6952626

[21] Freshwater ES , Crouch R. Technology for trauma: testing the validity of a smartphone app for pre-hospital clinicians. Int Emerg Nurs 2015; 23 (1): 32–7. doi: 10.1016/j.ienj.2014.04.003. 24837711

[22] Azad-Khaneghah P , Neubauer N, Miguel Cruz A, et al Mobile health app usability and quality rating scales: a systematic review. Disabil Rehabil Assist Technol 2021; 16 (7): 712–21. doi: 10.1080/17483107.2019.1701103. 31910687

[23] Ellsworth MA , Dziadzko M, O'Horo JC, et al An appraisal of published usability evaluations of electronic health records via systematic review. J Am Med Inform Assoc 2017; 24 (1): 218–26. doi: 10.1093/jamia/ocw046. 27107451 PMC7654077

[24] Muro-Culebras A , Escriche-Escuder A, Martin-Martin J, et al Tools for evaluating the content, efficacy, and usability of mobile health apps according to the consensus-based standards for the selection of health measurement instruments: systematic review. JMIR Mhealth Uhealth 2021; 9 (12): e15433. doi: 10.2196/15433. 34855618 PMC8686474

[25] Yáñez-Gómez R , Cascado-Caballero D, Sevillano J-L. Academic methods for usability evaluation of serious games: a systematic review. Multimed Tools Appl 2017; 76 (4): 5755–84. doi: 10.1007/s11042-016-3845-9.

[26] Page MM , Bossuyt PM, Boutron I, et al The PRISMA 2020 statement: an updated guideline for reporting systematic reviews. PLoS Med 2021; 18 (3): e1003583. 33780438 10.1371/journal.pmed.1003583 PMC8007028

[27] Wohlgemut J , Pisirir E. Usability of mobile clinical decision support systems designed for clinicians treating patients experiencing medical emergencies: a systematic review. PROSPERO 2021;CRD42021292014.

[28] Ouzzani M , Hammady H, Fedorowicz Z, et al Rayyan-a web and mobile app for systematic reviews. Syst Rev 2016; 5 (1): 210. doi: 10.1186/s13643-016-0384-4. 27919275 PMC5139140

[29] Downs SH , Black N. The feasibility of creating a checklist for the assessment of the methodological quality both of randomised and non-randomised studies of health care interventions. J Epidemiol Community Health 1998; 52 (6): 377–84. 9764259 10.1136/jech.52.6.377 PMC1756728

[30] Skivington K , Matthews L, Simpson SA, et al A new framework for developing and evaluating complex interventions: update of Medical Research Council guidance. BMJ 2021; 374: n2061. doi: 10.1136/bmj.n2061. 34593508 PMC8482308

[31] Booth A , Noyes J, Flemming K, Moore G, Tunçalp Ö, Shakibazadeh E. Formulating questions to address the acceptability and feasibility of complex interventions in qualitative evidence synthesis. BMJ Glob Health 2019; 4 (Suppl 1): e001107. 10.1136/bmjgh-2018-001107 PMC6350737 30775019

[32] Braun V , Clarke V. Using thematic analysis in psychology. Qual Res Psychol 2006; 3 (2): 77–101.

[33] Amin S , Gupta V, Du G, et al Developing and demonstrating the viability and availability of the multilevel implementation strategy for syncope optimal care through engagement (mission) syncope app: Evidence-based clinical decision support tool. J Med Internet Res 2021; 23 (11): e25192. doi: 10.2196/25192. 34783669 PMC8663445

[34] Gesell SB , Golden SL, Limkakeng AT Jr, et al Implementation of the HEART Pathway: Using the consolidated framework for implementation research. Crit Pathw Cardiol 2018; 17 (4): 191–200. doi: 10.1097/HPC.0000000000000154. 30418249 PMC6234854

[35] McCulloh RJ , Fouquet SD, Herigon J, et al Development and implementation of a mobile device-based pediatric electronic decision support tool as part of a national practice standardization project. J Am Med Inform Assoc 2018; 25 (9): 1175–82. doi: 10.1093/jamia/ocy069. 29889255 PMC6118866

[36] Schoemans HM , Goris K, Van Durm R, et al; EBMT Transplantation Complications Working Party. The eGVHD app has the potential to improve the accuracy of graft-versus-host disease assessment: a multicenter randomized controlled trial. Haematologica 2018; 103 (10): 1698–707. doi: 10.3324/haematol.2018.190777. 29903762 PMC6165809

[37] Schoemans H , Goris K, Durm RV, et al Development, preliminary usability and accuracy testing of the EBMT ‘eGVHD App’ to support GvHD assessment according to NIH criteria-a proof of concept. Bone Marrow Transplant 2016; 51 (8): 1062–5. doi: 10.1038/bmt.2016.26. 27042834

[38] Corazza F , Snijders D, Arpone M, et al Development and usability of a novel interactive tablet app (PediAppRREST) to support the management of pediatric cardiac arrest: Pilot high-fidelity simulation-based study. JMIR mHealth Uhealth 2020; 8 (10): e19070. doi: 10.2196/19070. 32788142 PMC7563631

[39] Barnes J , Duffy A, Hamnett N, et al The mersey burns app: evolving a model of validation. Emerg Med J 2015; 32 (8): 637–41. doi: 10.1136/emermed-2013-203416. 25371408

[40] Chang P , Tzeng Y-M, Wu S-C, Sang Y-Y, Chen S-S. Development and comparison of user acceptance of advanced comprehensive triage PDA support system with a traditional terminal alternative system. AMIA Annu Symp Proc 2003; 2003: 140–4. 14728150 PMC1479918

[41] Chang P , Hsu Y-S, Tzeng Y-M, Sang Y-Y, Hou I-C, Kao W-F. The development of intelligent, triage-based, mass-gathering emergency medical service pda support systems. J Nurs Res 2004; 12 (3): 227–36. doi: 10.1097/01.JNR.0000387506.06502.90. 15362014

[42] Clebone A , Strupp KM, Whitney G, et al; Pedi Crisis Application Working Group. Development and usability testing of the society for pediatric anesthesia pedi crisis mobile application. Anesth Analg 2019; 129 (6): 1635–44. doi: 10.1213/ANE.0000000000003935. 31743185

[43] Brooke J. SUS - a quick and dirty usability scale. Usability Eval Ind 1996; 194: 189–94.

[44] Laugwitz BH , Held T, Schrepp M. Construction and evaluation of a user experience questionnaire. In: Holzinger A, ed. USAB 2008: HCI and Usability for Education and Work. Springer; 2008: 63–76.

[45] NASA. NASA Task Load Index (TLX) Version 1.0 User's Guide. Moffett Field, CA: NASA Ames Research Center; 1985.

[46] Ellington LE , Najjingo I, Rosenfeld M, et al Health workers' perspectives of a mobile health tool to improve diagnosis and management of paediatric acute respiratory illnesses in Uganda: a qualitative study. BMJ Open 2021; 11 (7): e049708. doi: 10.1136/bmjopen-2021-049708. PMC8291301 34281930

[47] Frandes M , Timar B, Tole A, et al Mobile technology support for clinical decision in diabetic keto-acidosis emergency. Stud Health Technol Informatics 2015; 210: 316–20. 25991157

[48] Ginsburg AS , Delarosa J, Brunette W, et al mPneumonia: development of an innovative mHealth application for diagnosing and treating childhood pneumonia and other childhood illnesses in low-resource settings. PLoS One 2015; 10 (10): e0139625. doi: 10.1371/journal.pone.0139625. 26474321 PMC4608740

[49] Graber ML , Franklin N, Gordon R. Diagnostic error in internal medicine. Arch Intern Med 2005; 165 (13): 1493–9. 16009864 10.1001/archinte.165.13.1493

[50] Ginsburg AS , Tawiah Agyemang C, Ambler G, et al mPneumonia, an innovation for diagnosing and treating childhood pneumonia in low-resource settings: a feasibility, usability and acceptability study in Ghana. PLoS One 2016; 11 (10): e0165201. doi: 10.1371/journal.pone.0165201. 27788179 PMC5082847

[51] Bamidis PD , Konstantinidis ST, Rodrigues PP, eds. Design and Development of a Mobile Decision Support System: Guiding Clinicians Regarding Law in the Practice of Psychiatry in Emergency Department. In: Proceedings - IEEE Symposium on Computer-Based Medical Systems, Thessaloniki; Greece. Institute of Electrical and Electronics Engineers Inc; 2017: 67–72.

[52] Klingberg A , Wallis LA, Hasselberg M, et al Teleconsultation using mobile phones for diagnosis and acute care of burn injuries among emergency physicians: mixed-methods study. JMIR mHealth Uhealth 2018; 6 (10): e11076. doi: 10.2196/11076. 30341047 PMC6231743

[53] Yen P-Y , Wantland D, Bakken S. Development of a customizable health IT usability evaluation scale. AMIA Annu Symp Proc 2010; 2010: 917–21. 21347112 PMC3041285

[54] Klingberg A , Sawe HR, Hammar U, et al M-health for burn injury consultations in a low-resource setting: an acceptability study among health care providers. Telemed J E Health 2020; 26 (4): 395–405. doi: 10.1089/tmj.2019.0048. 31161967 PMC7187966

[55] Moore GC , Benbasat I. Development of an instrument to measure the perceptions of adopting an information technology innovation. Inf Syst Res 1991; 2 (3): 192–222.

[56] Hill RJ , Fishbein M, Ajzen I. Belief, attitude, intention and behavior: an introduction to theory and research. Contemp Sociol 1977; 6 (2): 244.

[57] O’Sullivan D , Doyle J, Michalowski W, Wilk S, Thomas R, Farion K. Expanding usability analysis with intrinsic motivation concepts to learn about CDSS adoption: a case study. Health Policy and Technology 2014; 3 (2): 113–25. doi: 10.1016/j.hlpt.2014.02.001.

[58] Paradis M , Stiell I, Atkinson KM, et al Acceptability of a mobile clinical decision tool among emergency department clinicians: development and evaluation of the Ottawa rules app. JMIR mHealth Uhealth 2018; 6 (6): e10263. doi: 10.2196/10263. 29891469 PMC6018230

[59] Parasuraman A , Colby CL. An updated and streamlined technology readiness index: TRI 2.0. J Serv Res 2015; 18 (1): 59–74. doi: 10.1177/1094670514539730.

[60] Quan AML , Stiell I, Perry JJ, et al Mobile clinical decision tools among emergency department clinicians: web-based survey and analytic data for evaluation of the Ottawa rules app. JMIR mHealth Uhealth 2020; 8 (1): e15503. doi: 10.2196/15503. 32012095 PMC7016628

[61] Rodríguez S , Sanz AM, Llano G, et al Acceptability and usability of a mobile application for management and surveillance of vector-borne diseases in Colombia: an implementation study. PLoS One 2020; 15 (5): e0233269. doi: 10.1371/journal.pone.0233269. 32469894 PMC7259752

[62] Agarwal S , LeFevre AE, Lee J, et al; WHO mHealth Technical Evidence Review Group. Guidelines for reporting of health interventions using mobile phones: mobile health (mHealth) evidence reporting and assessment (mERA) checklist. BMJ 2016; 352: i1174. 26988021 10.1136/bmj.i1174

[63] Grau I , Kostov B, Gallego JA, Grajales III F, Fernández-Luque L, Sisó-Almirall A. Assessment method for mobile health applications in Spanish: the iSYScore index. SEMERGEN - Med Fam 2016; 42 (8): 575–83. 10.1016/j.semerg.2015.12.001 26879598

[64] Stoyanov SR , Hides L, Kavanagh DJ, Zelenko O, Tjondronegoro D, Mani M. Mobile app rating scale: a new tool for assessing the quality of health mobile apps. JMIR Mhealth Uhealth 2015; 3 (1): e27. doi: 10.2196/mhealth.3422. 25760773 PMC4376132

[65] Stoyanov SR , Hides L, Kavanagh DJ, Wilson H. Development and validation of the user version of the mobile application rating scale (uMARS). JMIR Mhealth Uhealth 2016; 4 (2): e72. doi: 10.2196/mhealth.5849. 27287964 PMC4920963

[66] Schild S , Sedlmayr B, Schumacher A-K, Sedlmayr M, Prokosch H-U, St. Pierre M; German Cognitive Aid Working Group. A digital cognitive aid for anesthesia to support intraoperative crisis management: results of the user-centered design process. JMIR mHealth Uhealth 2019; 7 (4): e13226. doi: 10.2196/13226. 31033445 PMC6658227

[67] Lewis JR. Psychometric evaluation of the post-study system usability questionnaire: the PSSUQ. In: Proceedings of Human Factors Society Annual Meeting; 1992; 16: 1259–60. doi: 10.1177/154193129203601617.

[68] Schoemans HM , Goris K, Van Durm R, et al; Complications and Quality of Life Working Party of the EBMT. Accuracy and usability of the eGVHD app in assessing the severity of graft-versus-host disease at the 2017 EBMT annual congress. Bone Marrow Transplant 2018; 53 (4): 490–4. doi: 10.1038/s41409-017-0017-0. 29330389

[69] Sutham K , Khuwuthyakorn P, Thinnukool O. Thailand medical mobile application for patients triage base on criteria based dispatch protocol. BMC Med Inform Decis Mak 2020; 20 (1): 66. doi: 10.1186/s12911-020-1075-6. 32272928 PMC7147000

[70] Nielsen J. Enhancing the explanatory power of usability heuristics. In: Proceeding of ACM CHI’94 Conference, Boston, MA; 1994: 152–8.

[71] Yadav K , Chamberlain JM, Lewis VR, et al Designing real-time decision support for trauma resuscitations. Acad Emerg Med 2015; 22 (9): 1076–84. doi: 10.1111/acem.12747. 26300010 PMC5338692

[72] Yuan MJ , Finley GM, Long J, et al Evaluation of user interface and workflow design of a bedside nursing clinical decision support system. Interact J Med Res 2013; 2 (1): e4. doi: 10.2196/ijmr.2402. 23612350 PMC3628119

[73] Hajesmaeel-Gohari S , Khordastan F, Fatehi F, et al The most used questionnaires for evaluating satisfaction, usability, acceptance, and quality outcomes of mobile health. BMC Med Inform Decis Mak 2022; 22 (1): 22. doi: 10.1186/s12911-022-01764-2. 35081953 PMC8793175

[74] Yen P-Y , Bakken S. Review of health information technology usability study methodologies. J Am Med Inform Assoc 2012; 19 (3): 413–22. doi: 10.1136/amiajnl-2010-000020. 21828224 PMC3341772

[75] Kawamoto K , Houlihan CA, Balas EA, et al Improving clinical practice using clinical decision support systems: a systematic review of trials to identify features critical to success. BMJ 2005; 330 (7494): 765. doi: 10.1136/bmj.38398.500764.8F. 15767266 PMC555881

[76] Reisner AT , Khitrov MY, Chen L, et al Development and validation of a portable platform for deploying decision-support algorithms in prehospital settings. Appl Clin Inform 2013; 4 (3): 392–402. doi: 10.4338/ACI-2013-04-RA-0023. 24155791 PMC3799209

[77] Kappen TH , van Klei WA, van Wolfswinkel L, et al Evaluating the impact of prediction models: lessons learned, challenges, and recommendations. Diagn Progn Res 2018; 2: 11. doi: 10.1186/s41512-018-0033-6. 31093561 PMC6460651

[78] Gruppen LD , Wolf FM, Billi JE. Information gathering and integration as sources of error in diagnostic decision making. Med Decis Making 1991; 11 (4): 233–9. 1766327 10.1177/0272989X9101100401

[79] Loftus TJ , Tighe PJ, Filiberto AC, et al Artificial intelligence and surgical decision-making. JAMA Surg 2020; 155 (2): 148–58. doi: 10.1001/jamasurg.2019.4917. 31825465 PMC7286802

[80] Sendak M , Elish MC, Gao M, et al The human body is a black box. In: Proceedings of the 2020 Conference on Fairness, Accountability, and Transparency; 2020: 99–109.

[81] Neves MR , Marsh DWR. Modelling the impact of AI for clinical decision support. Artif Intell Med 2019; 11526: 292–7.

[82] Naismith LM , Cheung JJ, Ringsted C, et al Limitations of subjective cognitive load measures in simulation-based procedural training. Med Educ 2015; 49 (8): 805–14. doi: 10.1111/medu.12732. 26152492

[83] Norman DA. The Design of Everyday Things. Cambridge, MA: MIT Press; 2013.

[84] Gusenbauer M , Haddaway NR. Which academic search systems are suitable for systematic reviews or meta-analyses? Evaluating retrieval qualities of Google Scholar, PubMed, and 26 other resources. Res Synth Methods 2020; 11 (2): 181–217. doi: 10.1002/jrsm.1378. 31614060 PMC7079055

[85] Boeker M , Vach W, Motschall E. Google Scholar as replacement for systematic literature searches: good relative recall and precision are not enough. BMC Med Res Methodol 2013; 13: 131. 24160679 10.1186/1471-2288-13-131 PMC3840556

