# Supplementary material for: Methods used to evaluate usability of mobile clinical decision support systems for healthcare emergencies: a systematic review and qualitative synthesis
Source: JAMIA Open. 2023 Jul 12;6(3):ooad051. doi: 10.1093/jamiaopen/ooad051 (PMC10336299; doi:10.1093/jamiaopen/ooad051)
Supplement: ooad051_Supplementary_Data [file ooad051_supplementary_data.docx]

# SUPPLEMENTARY MATERIAL

Supplementary Table 1- Goals, associated questions, and metrics of the systematic review

| Goal | Question | Metric |
| --- | --- | --- |
| G1 – Determine the methods of usability evaluation used by researchers of mobile healthcare decision support in emergencies | RQ1.1 – What definition of Usability is used? | Definition of usability described by paper |
|  | RQ1.2 – What are the evaluation methods (quantitative and qualitative) used? | Evaluation method category and type used in paper |
|  | RQ1.3 – What usability metrics are being evaluated? | Evaluation metrics being tested |
|  | RQ1.4 – What is the number of users evaluated for each method? | Number of participants per method |
|  | RQ1.5 – What are the quantitative results for each method? | Statistical (or other) quantitative results |
|  | RQ1.6 – What are the qualitative results for each method? | Qualitative synthesis of usability-related barriers and facilitators of adoption according to study method |
| G2 – Determine characteristics of healthcare decision support in emergencies of included studies | RQ2.1- What medical specialty/setting would this be used in? | Number and type of medical specialties/settings targeted |
|  | RQ2.2- What is the intended patient condition which the app/tool/system addresses? | Number and type of patient conditions targeted |
|  | RQ2.3- What is the complexity of the app/tool/system? (nature of a) input, b) computation, c) output) | Input given to the app/tool/system  Computation performed by app/tool/system (prediction models, rule-based decision support)  Outputs of the app/tool/system |
|  | RQ2.4- What is the hardware/device the app is designed for? | The physical hardware/device/medium |

Supplementary Table 2- PRISMA statement[26]

| Section and Topic | Item # | Checklist item | Location where item is reported |
| --- | --- | --- | --- |
| TITLE | | |  |
| Title | 1 | Identify the report as a systematic review. | 1 |
| ABSTRACT | | |  |
| Abstract | 2 | See the PRISMA 2020 for Abstracts checklist. | 2 |
| INTRODUCTION | | |  |
| Rationale | 3 | Describe the rationale for the review in the context of existing knowledge. | 5-6 |
| Objectives | 4 | Provide an explicit statement of the objective(s) or question(s) the review addresses. | 6, Supp Table 1 |
| METHODS | | |  |
| Eligibility criteria | 5 | Specify the inclusion and exclusion criteria for the review and how studies were grouped for the syntheses. | 8, Table 1 |
| Information sources | 6 | Specify all databases, registers, websites, organisations, reference lists and other sources searched or consulted to identify studies. Specify the date when each source was last searched or consulted. | 7 |
| Search strategy | 7 | Present the full search strategies for all databases, registers and websites, including any filters and limits used. | 7, Supp Tables 3 & 4 |
| Selection process | 8 | Specify the methods used to decide whether a study met the inclusion criteria of the review, including how many reviewers screened each record and each report retrieved, whether they worked independently, and if applicable, details of automation tools used in the process. | 8-9 |
| Data collection process | 9 | Specify the methods used to collect data from reports, including how many reviewers collected data from each report, whether they worked independently, any processes for obtaining or confirming data from study investigators, and if applicable, details of automation tools used in the process. | 9-10 |
| Data items | 10a | List and define all outcomes for which data were sought. Specify whether all results that were compatible with each outcome domain in each study were sought (e.g. for all measures, time points, analyses), and if not, the methods used to decide which results to collect. | 9-10 (Supp Table 1) |
|  | 10b | List and define all other variables for which data were sought (e.g. participant and intervention characteristics, funding sources). Describe any assumptions made about any missing or unclear information. | 9-10 |
| Study risk of bias assessment | 11 | Specify the methods used to assess risk of bias in the included studies, including details of the tool(s) used, how many reviewers assessed each study and whether they worked independently, and if applicable, details of automation tools used in the process. | 9, Supp Table 5 |
| Effect measures | 12 | Specify for each outcome the effect measure(s) (e.g. risk ratio, mean difference) used in the synthesis or presentation of results. | 9-10, Supp Table 1 |
| Synthesis methods | 13a | Describe the processes used to decide which studies were eligible for each synthesis (e.g. tabulating the study intervention characteristics and comparing against the planned groups for each synthesis (item #5)). | 9-10 |
|  | 13b | Describe any methods required to prepare the data for presentation or synthesis, such as handling of missing summary statistics, or data conversions. | 9-10 |
|  | 13c | Describe any methods used to tabulate or visually display results of individual studies and syntheses. | 10 |
|  | 13d | Describe any methods used to synthesize results and provide a rationale for the choice(s). If meta-analysis was performed, describe the model(s), method(s) to identify the presence and extent of statistical heterogeneity, and software package(s) used. | 10 |
|  | 13e | Describe any methods used to explore possible causes of heterogeneity among study results (e.g. subgroup analysis, meta-regression). | NA |
|  | 13f | Describe any sensitivity analyses conducted to assess robustness of the synthesized results. | NA |
| Reporting bias assessment | 14 | Describe any methods used to assess risk of bias due to missing results in a synthesis (arising from reporting biases). | NA |
| Certainty assessment | 15 | Describe any methods used to assess certainty (or confidence) in the body of evidence for an outcome. | NA |
| RESULTS | | |  |
| Study selection | 16a | Describe the results of the search and selection process, from the number of records identified in the search to the number of studies included in the review, ideally using a flow diagram. | 11 and Figure 1 |
|  | 16b | Cite studies that might appear to meet the inclusion criteria, but which were excluded, and explain why they were excluded. | 11 and Figure 1 |
| Study characteristics | 17 | Cite each included study and present its characteristics. | 11-15, Tables 2, Supp Tables 7 & 8 |
| Risk of bias in studies | 18 | Present assessments of risk of bias for each included study. | 15, Figures 2 & 3 |
| Results of individual studies | 19 | For all outcomes, present, for each study: (a) summary statistics for each group (where appropriate) and (b) an effect estimate and its precision (e.g. confidence/credible interval), ideally using structured tables or plots. | Figures 4, Tables 3 and 4, Supp Table 9 |
| Results of syntheses | 20a | For each synthesis, briefly summarise the characteristics and risk of bias among contributing studies. | 15-17, Figure 3 |
|  | 20b | Present results of all statistical syntheses conducted. If meta-analysis was done, present for each the summary estimate and its precision (e.g. confidence/credible interval) and measures of statistical heterogeneity. If comparing groups, describe the direction of the effect. | NA |
|  | 20c | Present results of all investigations of possible causes of heterogeneity among study results. | NA |
|  | 20d | Present results of all sensitivity analyses conducted to assess the robustness of the synthesized results. | NA |
| Reporting biases | 21 | Present assessments of risk of bias due to missing results (arising from reporting biases) for each synthesis assessed. | NA |
| Certainty of evidence | 22 | Present assessments of certainty (or confidence) in the body of evidence for each outcome assessed. | NA |
| DISCUSSION | | |  |
| Discussion | 23a | Provide a general interpretation of the results in the context of other evidence. | 20-23 |
|  | 23b | Discuss any limitations of the evidence included in the review. | 22-23 |
|  | 23c | Discuss any limitations of the review processes used. | 22-23 |
|  | 23d | Discuss implications of the results for practice, policy, and future research. | 20-22 |
| OTHER INFORMATION | | |  |
| Registration and protocol | 24a | Provide registration information for the review, including register name and registration number, or state that the review was not registered. | 3 and 7 |
|  | 24b | Indicate where the review protocol can be accessed, or state that a protocol was not prepared. | 3 and 7 |
|  | 24c | Describe and explain any amendments to information provided at registration or in the protocol. | NA |
| Support | 25 | Describe sources of financial or non-financial support for the review, and the role of the funders or sponsors in the review. | 24 |
| Competing interests | 26 | Declare any competing interests of review authors. | 24 |
| Availability of data, code and other materials | 27 | Report which of the following are publicly available and where they can be found: template data collection forms; data extracted from included studies; data used for all analyses; analytic code; any other materials used in the review. | 10 |

## Supplementary Table 3- Search terms

| Combine with OR | Combine with OR | Combine with OR | Combine with OR | Combine with OR | Combine with OR | Combine with OR |
| --- | --- | --- | --- | --- | --- | --- |
| Usability* | Assess | Mobile | Application | Decision support | Healthcare | Emergency |
| Ease of use | Assessment | Smartphone | App | Decision aid | Hospital | Emergencies |
| Usefulness | Test | Tablet | Program | Decision tool | Medical | Urgent |
| Utility | Testing | Digital | Software | Risk prediction | Medicine | Acute |
|  | Evaluate | Electronic | System | Prognosis | Surgical | Crisis |
|  | Evaluation | Handheld | Technology | Diagnosis | Surgery |  |
|  |  | Portable | Website | Decision making | Clinical |  |
|  |  |  | Device | Decision-making | Prehospital |  |
|  |  |  |  | Guideline | Pre-hospital |  |
|  |  |  |  | Protocol | eHealth |  |
|  |  |  |  |  | mHealth |  |
| *There are many other terms related to usability, such as learnability/learnable/easy to learn, acceptability/acceptable, and likability/likable. However, we limited the search to the concepts of usability and usefulness according to the TAM framework, and thus searched for “usability” and its main synonym (ease of use), and “usefulness” and its main synonym (utility) [6,11] | | | | | | |

## Supplementary Table 4- Search strings for Pubmed/Medline, Embase, Scopus and IEEE Xplore

| Search Database | Search String used |
| --- | --- |
| Pubmed | (("usability"[Title/Abstract] OR "ease of use"[Title/Abstract] OR "usefulness"[Title/Abstract] OR "utility"[Title/Abstract]) AND ("Assess"[Title/Abstract] OR "Assessment"[Title/Abstract] OR "test"[Title/Abstract] OR "testing"[Title/Abstract] OR "evaluation"[Title/Abstract] OR "evaluate"[Title/Abstract]) AND ("mobile"[Title/Abstract] OR "smartphone"[Title/Abstract] OR "tablet"[Title/Abstract] OR "digital"[Title/Abstract] OR "electronic"[Title/Abstract] OR "handheld"[Title/Abstract] OR "portable"[Title/Abstract]) AND ("application"[Title/Abstract] OR "app"[Title/Abstract] OR "program"[Title/Abstract] OR "software"[Title/Abstract] OR "system"[Title/Abstract] OR "technology"[Title/Abstract] OR "website"[Title/Abstract] OR "device"[Title/Abstract]) AND ("decision support"[Title/Abstract] OR "decision aid"[Title/Abstract] OR "decision tool"[Title/Abstract] OR "risk prediction"[Title/Abstract] OR "prognosis"[Title/Abstract] OR "diagnosis"[Title/Abstract] OR "decision making"[Title/Abstract] OR "decision-making"[Title/Abstract] OR "guideline"[Title/Abstract] OR "protocol"[Title/Abstract]) AND ("healthcare"[Title/Abstract] OR "hospital"[Title/Abstract] OR "medical"[Title/Abstract] OR "medicine"[Title/Abstract] OR "surgical"[Title/Abstract] OR "surgery"[Title/Abstract] OR "clinical"[Title/Abstract] OR "prehospital"[Title/Abstract] OR "pre-hospital"[Title/Abstract] OR "eHealth"[Title/Abstract] OR "mHealth"[Title/Abstract] OR "e-Health"[Title/Abstract] OR "m-Health"[Title/Abstract]) AND ("emergency"[Title/Abstract] OR "emergencies"[Title/Abstract] OR "urgent"[Title/Abstract] OR "acute"[Title/Abstract] OR "crisis"[Title/Abstract])) |
| Embase | (usability:ab,ti OR 'ease of use':ab,ti OR usefulness:ab,ti OR utility:ab,ti) AND (assess:ab,ti OR assessment:ab,ti OR test:ab,ti OR testing:ab,ti OR evaluate:ab,ti OR evaluation:ab,ti) AND (mobile:ab,ti OR smartphone:ab,ti OR tablet:ab,ti OR digital:ab,ti OR electronic:ab,ti OR 'handheld':ab,ti OR 'portable':ab,ti) AND (application:ab,ti OR app:ab,ti OR program:ab,ti OR software:ab,ti OR system:ab,ti OR technology:ab,ti OR website:ab,ti OR device:ab,ti) AND ('decision support':ab,ti OR 'decision aid':ab,ti OR 'decision tool':ab,ti OR 'risk prediction':ab,ti OR prognosis:ab,ti OR diagnosis:ab,ti OR 'decision making':ab,ti OR 'decision-making':ab,ti OR guideline:ab,ti OR protocol:ab,ti) AND (healthcare:ab,ti OR hospital:ab,ti OR medical:ab,ti OR medicine:ab,ti OR surgical:ab,ti OR surgery:ab,ti OR clinical:ab,ti OR prehospital:ab,ti OR pre-hospital:ab,ti OR eHealth:ab,ti OR mHealth:ab,ti) AND (emergency:ab,ti OR emergencies:ab,ti OR urgent:ab,ti OR acute:ab,ti OR crisis:ab,ti) |
| Scopus | TITLE-ABS-KEY (("usability" OR "ease of use" OR "usefulness" OR "utility") AND ("Assess" OR "Assessment" OR "test" OR “testing” OR "evaluation" OR "evaluate") AND ("mobile" OR "smartphone" OR “tablet” OR “digital” OR “electronic” OR “handheld” OR “portable”) AND ("application" OR "app" OR “program” OR "software" OR “system” OR “technology” OR “website” OR “device”) AND (“decision support” OR “decision aid” OR “decision tool” OR “risk prediction” OR “prognosis” OR “diagnosis” OR “decision making” OR “decision-making” OR "protocol" OR "guideline") AND ("healthcare" OR "hospital" OR "medical" OR "medicine" OR "surgical" OR "surgery" OR "clinical" OR “prehospital” OR “pre-hospital” OR “eHealth” OR “mHealth”) AND ("emergency" OR "emergencies" OR "urgent" OR “acute” OR “crisis”)) |
| IEEE Xplore* | ("Abstract":"usability" OR "Abstract":"ease of use" OR "Abstract":"usefulness" OR "Abstract":"utility") AND ("Abstract":"Assess" OR "Abstract":"Assessment" OR "Abstract":"test" OR "Abstract":“testing” OR "Abstract":"evaluation" OR "Abstract":"evaluate") AND ("Abstract":"application" OR "Abstract":"app" OR "Abstract":“program” OR "Abstract":"software" OR "Abstract":“system” “technology” OR "Abstract":“website” OR "Abstract":“device”) AND ("Abstract":“decision support” OR "Abstract":“decision aid” OR "Abstract":“decision tool” OR "Abstract":“risk prediction” OR "Abstract":“prognosis” OR "Abstract":“diagnosis” OR "Abstract":“decision making” OR "Abstract":“decision-making” OR "Abstract":"protocol" OR "Abstract":"guideline") AND ("Abstract":"healthcare" OR "Abstract":"hospital" OR "Abstract":"medical" OR "Abstract":"medicine" OR "Abstract":"surgical" OR "Abstract":"surgery" OR "Abstract":"clinical" OR "Abstract":“prehospital” OR "Abstract":“pre-hospital” OR "Abstract":“eHealth” OR "Abstract":“mHealth”) AND ("Abstract":"emergency" OR "Abstract":"emergencies" OR "Abstract":"urgent" OR "Abstract":“acute” OR "Abstract":“crisis”) AND ("Abstract":"mobile" OR "Abstract":"smartphone" OR "Abstract":“tablet” OR "Abstract":“digital” OR "Abstract":“electronic” OR "Abstract":“handheld” OR "Abstract":“portable”) |
|  | *(searching IEEE Xplore for titles revealed 0 search results. There was no option to search both abstracts and titles simultaneously) |

## Supplementary Table 5- Modified Downs and Black checklist[29]

| Reporting | | |
| --- | --- | --- |
| 1 | Is the hypothesis/aim/objective of the study clearly described? | Yes/no |
| 2 | Are the main outcomes to be measured clearly described in the Introduction or Methods section? (If the main outcomes are first mentioned in the Results section, the question should be answered no) | Yes/no |
| 3 | Are the characteristics of the patients included in the study clearly described ? (In cohort studies and trials, inclusion and/or exclusion criteria should be given. In case-control studies, a case-definition and the source for controls should be given.) | Yes/no |
| 4 | Are the interventions of interest clearly described? (Treatments and placebo (where relevant) that are to be compared should be clearly described.) | Yes/no |
| 6 | Are the main findings of the study clearly described? (Simple outcome data (including denominators and numerators) should be reported for all major findings so that the reader can check the major analyses and conclusions. (This question does not cover statistical tests which are considered below). | Yes/no |
| 7 | Does the study provide estimates of the random variability in the data for the main outcomes? (In non normally distributed data the inter-quartile range of results should be reported. In normally distributed data the standard error, standard deviation or confidence intervals should be reported. If the distribution of the data is not described, it must be assumed that the estimates used were appropriate and the question should be answered yes.) | Yes/no |
| 8 | Have all important adverse events that may be a consequence of the intervention been reported? (This should be answered yes if the study demonstrates that there was a comprehensive attempt to measure adverse events. (A list of possible adverse events is provided).) | Yes/no |
| 10 | Have actual probability values been reported(e.g. 0.035 rather than <0.05) for the main outcomes except where the probability value is less than 0.001? | Yes/no |
| External Validity | | |
| 11 | Were the subjects asked to participate in the study representative of the entire population from which they were recruited? (The study must identify the source population for patients and describe how the patients were selected. Patients would be representative if they comprised the entire source population, an unselected sample of consecutive patients, or a random sample. Random sampling is only feasible where a list of all members of the relevant population exists. Where a study does not report the proportion of the source population from which the patients are derived, the question should be answered as unable to determine.) | Yes/no/unable to determine |
| 13 | Were the staff, places, and facilities where the patients were treated, representative of the treatment the majority of patients receive? For the question to be answered yes the study should demonstrate that the intervention was representative of that in use in the source population. The question should be answered no if, for example, the intervention was undertaken in a specialist centre unrepresentative of the hospitals most of the source population would attend. | Yes/no/unable to determine |
| Internal Validity: Bias | | |
| 15 | Was an attempt made to blind those measuring the main outcomes of the intervention? | Yes/no/unable to determine |
| 16 | If any of the results of the study were based on “data dredging”, was this made clear? Any analyses that had not been planned at the outset of the study should be clearly indicated. If no retrospective unplanned subgroup analyses were reported, then answer yes. | Yes/no/unable to determine |
| 18 | Were the statistical tests used to assess the main outcomes appropriate? The statistical techniques used must be appropriate to the data. For example nonparametric methods should be used for small sample sizes. Where little statistical analysis has been undertaken but where there is no evidence of bias, the question should be answered yes. If the distribution of the data (normal or not) is not described it must be assumed that the estimates used were appropriate and the question should be answered yes. | Yes/no/unable to determine |
| 19 | Was compliance with the intervention/s reliable? Where there was non compliance with the allocated treatment or where there was contamination of one group, the question should be answered no. For studies where the effect of any misclassification was likely to bias any association to the null, the question should be answered yes. | Yes/no/unable to determine |
| 20 | Were the main outcome measures used accurate (valid and reliable)? For studies where the outcome measures are clearly described, the question should be answered yes. For studies which refer to other work or that demonstrates the outcome measures are accurate, the question should be answered as yes. | Yes/no/unable to determine |
| Internal Validity: Confounding (selection bias) | | |
| 21 | Were the patients in different intervention groups (trials and cohort studies) or were the cases and controls (case-control studies) recruited from the same population? For example, patients for all comparison groups should be selected from the same hospital. The question should be answered unable to determine for cohort and case control studies where there is no information concerning the source of patients included in the study. | Yes/no/unable to determine |
| 22 | Were study subjects in different intervention groups (trials and cohort studies) or were the cases and controls (case-control studies) recruited over the same period of time? For a study which does not specify the time period over which patients were recruited, the question should be answered as unable to determine. | Yes/no/unable to determine |
| 23 | Were study subjects randomised to intervention groups? Studies which state that subjects were randomised should be answered yes except where method of randomisation would not ensure random allocation. For example alternate allocation would score no because it is predictable. | Yes/no/unable to determine |
| 24 | Was the randomised intervention assignment concealed from both patients and health care staff until recruitment was complete and irrevocable? All non-randomised studies should be answered no. If assignment was concealed from patients but not from staff, it should be answered no. | Yes/no/unable to determine |
| Downs and Black checklist questions 5, 9, 12, 14, 17, 25, 26, and 27 were omitted. | | |

## Supplementary Table 6- PerSPecTIF question formulation framework for qualitative evidence syntheses[31]

| Per | S | P | E | (C) | Ti | F |
| --- | --- | --- | --- | --- | --- | --- |
| Perspective | Setting | Phenomenon of interest/ Problem | Environment | Comparison (optional) | Time/ Timing | Findings |
| From the perspective of academics designing and testing Mobile CDSSs | Mobile CDSSs used or tested in medical emergency setting | What is the usability of mobile CDSSs | Within an environment of clinicians making decisions for patients experiencing medical emergencies | Some compare with existing system, or paper document, or no comparison | During the medical emergency (not before or after) | In relation to the clinician participants’ perceptions of CDSS usability |

Supplementary Table 7- Characteristics of mobile CDSS in healthcare emergencies which underwent usability evaluation in included studies

| Characteristic | n | % |
| --- | --- | --- |
| Geographical region of study conduct |  |  |
| Europe  North America  Africa  Asia  South America | 8  6  5  3  1 | 35  26  22  13  4 |
| Year of publication  2017-2021  2012-2016  2007-2011  2002-2006 | 13  8  0  2 | 57  35  0  9 |
| Study design  Observational  Controlled non-randomized  Randomized controlled trial | 20  2  1 | 87  9  4 |
| Methods category used  Questionnaire  User testing  Interview  Heuristic evaluation | 20  17  6  3 | 87  74  26  13 |
| Number of method categories used  Two  One  Three | 11  6  6 | 48  26  26 |
| Type of methods used  Only quantitative  Only qualitative  Both quantitative and qualitative | 10  1  12 | 43  4  52 |
| Validated methods  SUS[43]  TAM[6]  Nielsen’s Heuristics[70]  NASA-TLX[45]  TRI[59]  PSSUQ[67]  Other validated method*  Studies with no validated methods | 5  5  3  2  2  2  8  5 | 22  22  13  9  9  9  35  22 |
| Participants  Clinicians  Data managers  Usability engineers  Information scientists | 23  3  1  1 | 100  13  4  4 |
| Number of participants (median, IQR)  Questionnaire [n=20, missing=0]  User testing [n=17, missing=0]  Interview [n=6, missing=0]  Heuristic evaluation [n=3, missing=0] | 29  28  26  4 | 12-51  9-44  11-43  4-8 |
| Conditions  Multiple  Burns  Graft versus host disease  Pediatric respiratory illness  Other | 9  3  3  3  5 | 39  13  13  13  22 |
| Device  Mobile tablet  Mobile smartphone  Desktop web app  Mobile PDA  Laptop | 13  13  5  2  1 | 57  57  22  9  4 |
| Guideline on which CDSS is based  CDSS based on a guideline  CDSS not based on a guideline | 20  3 | 87  13 |
| Stage(s) of CDSS  Development  Feasibility  Evaluation  Implementation | 14  20  5  2 | 61  87  22  9 |
| Input type  Checkboxes / radio buttons  Number / text  Image  Automatic monitor and physical input | 18  8  3  2 | 78  35  13  9 |
| Output type  Text output  Numerical output  Image  Video | 22  10  2  1 | 96  43  9  4 |
| Output information  Recommendations  Specific treatment  Score, risk level or likelihood of diagnosis | 18  6  6 | 78  26  26 |
| *Others include UEQ[44], Health-ITUES[53], IDT[55], TPB[56], mERA[62], iSYScore index[63], MARS[64], uMARS[65] | | |

Supplementary Table 8- Mobile application characteristics per study

| Year | Author and Reference |  |  | Input | | Output | | |
| --- | --- | --- | --- | --- | --- | --- | --- | --- |
|  |  | Device | Name of system/  app | # |  | # | Type | Information |
| 2015 | Barnes[39] | Mobile (smartphone, tablet) | Mersey Burns App | 5 | Radio buttons/checkboxes; number/text inputs; image input | 1 | Text | Recommendations |
| 2003 | Chang[40] | Mobile (PDA) | NA | 293 | Radio buttons/checkboxes; number/text inputs | 1 | Numerical | Score |
| 2004 | Chang[41] | Mobile (PDA) | NA | 180 | Radio buttons/checkboxes; number/text inputs | 1 | Text | Recommendations |
| 2019 | Clebone[42] | Mobile (smartphone) | Pedi Crisis 2.0 App | 2 | Radio buttons/checkboxes; number/text inputs | 26 | Text | Treatment; recommendations |
| 2020 | Corazza[38] | Mobile (tablet) | PediARREST App | NA | Radio buttons/checkboxes; number/text inputs | 2 | Text; images | Recommendations |
| 2021 | Ellington[46] | Mobile (smartphone) | ALRITE | NA | NA | 3 | Text; images; videos | Recommendations |
| 2015 | Frandes[47] | Mobile (smartphone, tablet) | mDKA | NA | NA | NA | Numerical; text | Recommendations |
| 2015 | Ginsburg[48] | Mobile (tablet) | mPneumonia | NA | Automated monitor input; physical input | 2 | Text | Treatment; recommendations |
| 2016 | Ginsburg[49 ,50] | Mobile (tablet) | mPneumonia | NA | Automated monitor input; physical input | 2 | Text | Treatment; recommendations |
| 2017 | Khodambashi[51] | Mobile (smartphone, tablet) | NA | NA | Radio buttons/checkboxes | NA | Text | Treatment; recommendations |
| 2018 | Klingberg[52] | Mobile (smartphone) | Vula App | 13 | Radio buttons/checkboxes; number/text inputs; image input | 1 | Numerical; text | Recommendations |
| 2020 | Klingberg[54] | Mobile (smartphone) | Vula App | NA | Radio buttons/checkboxes; number/text inputs; image input | 1 | Numerical; text | Recommendations |
| 2014 | O'Sullivan[57] | Mobile (tablet); Desktop (web app) | MET3-AE | NA | Radio buttons/checkboxes | 2 | Numerical; text | Score; risk level |
| 2018 | Paradis[58] | Mobile (smartphone, tablet) | Ottawa Rules App | NA | Radio buttons/checkboxes | 3 | Text | Treatment; recommendations |
| 2020 | Quan[60] | Mobile (smartphone, tablet) | Ottawa Rules App 3.0.2 | NA | NA | 6 | Text | Treatment; recommendations |
| 2020 | Rodriguez[61] | Mobile (smartphone) | FeverDx | 22 | Radio buttons/checkboxes | 4 | Text | Recommendations |
| 2019 | Schild[66] | Mobile (tablet); Desktop (web app) | NA | NA | Radio buttons/checkboxes | NA | Text | Recommendations |
| 2016 | Schoemans[37] | Desktop (web app) | eGVHD App | 78 | Radio buttons/checkboxes | 1 | Numerical; text | Likelihood of diagnosis |
| 2018 | Schoemans[36] | Mobile (smartphone, tablet); Desktop (web app) | eGVHD App | 78 | Radio buttons/checkboxes | 1 | Numerical; text | Likelihood of diagnosis |
| 2018 | Schoemans[68] | Mobile (smartphone, tablet, laptop) | NA | 78 | Radio buttons/checkboxes | 1 | Numerical; text | Likelihood of diagnosis |
| 2020 | Sutham[69] | Mobile (smartphone) | Triagist App | NA | Radio buttons/checkboxes; number/text inputs | 1 | Numerical; text | Recommendations |
| 2015 | Yadav[71] | Desktop (web app) | NA | 10 | Radio buttons/checkboxes | 3 | Numerical; text | Risk level; recommendations |
| 2013 | Yuan[72] | Mobile (tablet) | NA | NA | Radio buttons/checkboxes | 10 | Text | Outcomes; recommendations |

## Supplementary Table 9- Quantitative results from included studies (n=22/23)

| Year | First Author and Reference | Validated Method used | Result Meaning/Interpretation |
| --- | --- | --- | --- |
| 2015 | Barnes[39] | NA | Q: User preferred App in emergency setting, had confidence in output, App was accurate, fast, ease of calculation, use and shading. U: App was time-saving vs paper form. |
| 2003 | Chang[40] | TAM[6] | Q: PDA had worse ease of use and willingness to accept than Terminal system by users. |
| 2004 | Chang[41] | TAM[6] | Q: Though more than half of users perceived the PDA systems to be easy to use and useful at mass gatherings, physicians found it less useful than nurses. |
| 2019 | Clebone[42] | SUS[43] | Q: SUS score was acceptable (83.4). U: Most participants maneuvered through the system efficiently and effectively. |
| 2020 | Corazza[38] | UEQ[44]; NASA-TLX[45] | Q: App had acceptable usability, though perceived workload was comparable whether app was used or not.  U: Using the app increased time to epinephrine. Team clinical performance was comparable whether app was used or not. App had trend of improving CPR, epinephrine dosing, and identifying reversible causes of cardiac arrest. |
| 2015 | Frandes[47] | NA | Q: Despite good average usability on questionnaire, only 66% users said they would use the mDKA app. |
| 2015 | Ginsburg[48] | SUS[43] | Q: Usability was acceptable for mPneumonia (SUS = 70). U: Time to complete that task varied widely between users, and many errors were identified. |
| 2016 | Ginsburg[49 ,50] | SUS[43] | Q: Users showed unanimous willingness to use the app. I: There was enthusiasm among users that it could be feasible, easy to use, could be learned quickly, though only two thirds thought it would be faster than paper. |
| 2017 | Khodambashi[51] | SUS[43] | Q: Usability was acceptable-excellent (SUS = 90). U: All users could perform all tasks effectively and efficiently. |
| 2018 | Klingberg[52] | Health-ITUES[53] | Q: Usability was rated highly, except for user control. U: Task analysis identified issues with navigation, meaning of icons and terminology, visibility of system status, consistency, and lack of user instructions. |
| 2020 | Klingberg[54] | TAM[6], IDT[55], TPB[56] | Q: Some usability aspects rated highly (≥6/7): ease of use, usefulness, compatibility, and attitude toward using the technology; while voluntariness and self-efficacy rated poorly (≤4/7). |
| 2014 | O'Sullivan[57] | NA | Q: Ease of use for the digital pen was poor, marred by variable user intrinsic motivation. Overall usability with the MET3-AE system was moderate, with most users finding it quick, easy to navigate, intuitive with good functionality. |
| 2018 | Paradis[58] | TRI[59] | Q: Two thirds of users found the app useful, and would continue using it, while three quarters would recommend it to colleagues. Technology readiness among participants was moderate. U: The most frequently used aspects were the TOH guidelines/algorithms, followed by the C-spine, Ankle and Knee rules. |
| 2020 | Quan[60] | TRI[59] | Q: Over two thirds found the app helpful in carrying out clinical rules, three quarters would recommend the app to colleagues, and 84% would keep using it. The C-spine rule was the most useful. Younger users were more ready for technology. U: The largest user group were nurses. 40% of app uses accessed the newly added rules (CCHR, TIA score and SAH rules). |
| 2020 | Rodriguez[61] | mERA[62], iSYScore index[63], MARS[64]; uMARS[65] | Q: The app was deemed to have good impact, functionality, would increase knowledge, and users demonstrated intention to change. |
| 2019 | Schild[66] | SUS[43] | Q: Usability was acceptable in the first prototype (SUS = 74), which improved slightly in the third prototype (SUS = 77). U: Users reported acceptable features including concept, design, function, intuitiveness, structure and ease of use. However there were usability problems identified to be addressed, including button size, absent buttons, confusing navigation, necessary training, and excessive clicks/tabs. |
| 2016 | Schoemans[37] | TAM[6], PSSUQ[67] | Q: eGVHD App achieved good usability according to PSSUQ, across several domains and overall.  U: eGVHD App had significantly improved diagnostic accuracy compared to using paper forms. |
| 2018 | Schoemans[36] | TAM[6], PSSUQ[67] | Q: eGVHD App achieved good usability according to PSSUQ, across several domains and overall.  U: eGVHD App had improved diagnostic accuracy compared to using paper forms. |
| 2018 | Schoemans[68] | NA | Q: The vast majority of those who used the eGVHD app found it reliable, would improve their diagnostic accuracy and would use it in practice, most on a desktop computer or mobile phone rather than tablet. U: Using eGVHD app significantly increased diagnostic accuracy compared to self-assessment, though was incorrect in 7% for acute GVHD and 2% for chronic GVHD, even with the app. This benefit was the same regardless of whether the user is a doctor or not. |
| 2020 | Sutham[69] | Nielsen’s Heuristics[70] | U: The application was shown to be useful in the majority of the scenarios in terms of operational time and reliability, but it is more likely to be suitable for use with non-trauma patients. |
| 2015 | Yadav[71] | Nielsen’s Heuristics[70] | U: Iterative improvements of eCDS improved user rating, but some barriers to usability remained.  H: Usability evaluation identified issues in every heuristic category. |
| 2013 | Yuan[72] | NASA TLX[45], Nielsen's Heuristics[70] | Q: The mental effort required to use the CDSS was reasonable. U: Users completed tasks within reasonable time frame.  H: Multiple heuristic violations were identified in each of 10 categories, with varying severity. |
| Ellington[46] did not report any quantitative results. Q, U, I, H are questionnaire, user-trial, interview, and heuristic studies, respectively; NA= not applicable; TAM= technology acceptance model; SUS= system usability scale; UEQ= user experience questionnaire; NASA TLX= National Aeronautics and Space Administration task load index; Health-ITUES= health information technology usability evaluation scale; IDT= innovation diffusion theory; TPB= theory of planned behavior; mERA= mobile health evidence reporting and assessment checklist; MARS= mobile application rating scale; uMARS= user version of the mobile application rating scale; PSSUQ= post-study system usability questionnaire; TRI= technology readiness index; CI= confidence interval; OR= odds ratio; PDA= personal digital assistant; IQR= inter-quartile range; SD= standard deviation; CPR= cardio-pulmonary resuscitation; ED= emergency department; CDSS= clinical decision support system; TOH= The Ottawa Hospital; CCHR= Canadian CT Head Rule; TIA= transient ischemic attack; SAH= sub-arachnoid haemorrhage. | | | |
